# Research on a financial fraud identification model by fusing a convolutional neural network

**DOI:** 10.1371/journal.pone.0348569

**Published:** 2026-05-22

**Authors:** Haiyan Lu, Shuhe Zhu, Yajing Zhang, Ruyi Lv

**Affiliations:** Department of accounting, Lanzhou University of Finance and Economics, Lanzhou, Gansu, China; University of Hamburg: Universitat Hamburg, GERMANY

## Abstract

To address the challenges of low accuracy and poor real-time performance in existing financial fraud identification methods for listed companies, this paper proposes a hybrid identification model (CNN-SVM) that fuses a convolutional neural network with a support vector machine. Utilizing a comprehensive dataset of 7,429 samples from non-financial A-share listed companies penalized for fraud between 2007 and 2022, the study employs random oversampling to rebalance the minority class, resulting in a robust training set of 13,540 samples. The model leverages a CNN architecture to automatically extract high-level features from 87 indicators spanning corporate governance, financial oversight, and operational metrics, which are then classified using a linear-kernel SVM. Experimental results demonstrate that the CNN-SVM model achieves a qualitative leap in performance, yielding an AUC of 0.97, a recall of 0.99, and an F1-score of 0.97, significantly outperforming traditional logistic regression and random forest benchmarks. These findings suggest that the proposed framework effectively balances training efficiency with high precision, providing a superior tool for real-time financial risk control.

## 1. Introduction

Financial fraud remains a major threat to capital market integrity, investor protection, and the efficient allocation of financial resources. By distorting financial statements and related disclosures, fraudulent reporting can mislead investors, weaken market confidence, and undermine regulatory effectiveness [1]. Under increasing market pressure and regulatory scrutiny, some firms may still engage in opportunistic manipulation of financial reporting. Such misconduct often takes the form of earnings inflation, false disclosure, asset misstatement, or other forms of material financial misrepresentation. In the Chinese listed-company context, fraud incentives are shaped by firm-level operating pressure, financing needs, governance weaknesses, and the institutional environment. Typical forms of fraud include fabricated transactions, misleading statements, profit inflation, and disclosure violations [2], all of which are difficult to identify accurately through manual external screening alone. Traditional manual identification techniques are increasingly inadequate for large-scale financial data analysis, which highlights the need for more efficient and accurate data-driven detection models.

Recent advances in machine learning (ML), together with increased computational capacity, have accelerated the adoption of data-driven methods for financial fraud detection in both research and practice. Previous studies have explored a variety of machine learning techniques for detecting financial statement fraud and related forms of financial misconduct. These methods have improved predictive performance by uncovering nonlinear patterns and interactions in financial data [3]. Among existing approaches, Logistic Regression (LR) remains an important benchmark because of its interpretability and widespread use in fraud-related classification research [4]. However, LR primarily serves as a linear baseline and may not fully capture complex nonlinear interactions among fraud-related variables. Although LR can be applied to both binary and multiclass settings, its representational capacity remains limited in high-dimensional fraud-detection tasks. Notably, Kim et al. [5] extended LR to a multinomial setting, but this line of work still relies mainly on manually specified feature relationships. Beyond LR, support vector machines, random forests, Extreme Gradient Boosting (XGBoost), and related algorithms have also been introduced into fraud detection and have shown the value of nonlinear classification on structured financial data [6]. Zhou et al. [7] introduced the X score framework for financial fraud identification and demonstrated the predictive value of carefully designed indicator systems. LuQ et al. [8] compared multiple machine-learning methods and showed that XGBoost can achieve strong predictive performance, whereas GAN-based approaches offer a promising direction for representation learning; however, these methods still face challenges related to feature dependence, interpretability, and robustness across settings. Taken together, these studies confirm the promise of machine learning in financial fraud detection, but they also indicate that methodological improvements are still needed for high-dimensional, imbalanced, and practically deployable fraud screening.

Although machine learning methods have shown promise in detecting corporate fraud, the field still lacks sufficiently standardized modeling pipelines and evaluation strategies for highly imbalanced listed-company fraud data [8]. Major challenges include complex nonlinear relationships, heterogeneous high-dimensional inputs, evolving fraud patterns, and the trade-off between predictive performance and computational efficiency. Accordingly, some studies have attempted to improve fraud detection through kernel design, model integration, and boosting-based optimization. For instance, Cecchini et al. [9] combined Support Vector Machines (SVM) with financial kernels to develop the Support Vector Machines-Financial Kernel (SVM-FK) model. Cheng Xuejiao [10] and Zhang Jiajia [11] each analyzed decision trees, Support Vector Machines, and neural networks, and proposed integrated identification models to improve recognition rates and reliability. Wu [12] established a recognition framework and compared the prediction results of the Light Gradient Boosting Machine (LightGBM) model with those of traditional models. Song et al. [13] proposed an ensemble model based on four different machine learning approaches, which demonstrated a recall ratio at least 10% higher than that of single models.

Other studies have focused on preprocessing strategies, especially class-imbalance handling, to improve minority-class detection performance. Perols [14] utilized the Support Vector Machine (SVM) model to propose three data sampling techniques, which enhanced the model’s efficiency in fraud detection. Cheng [15] applied an oversampling algorithm to address class imbalance, thereby improving the model’s performance on imbalanced datasets. Although ensemble methods can improve predictive performance, they may also increase computational complexity and reduce transparency in practical deployment.

Given the limitations of shallow and manually structured models, there is growing interest in whether deep-learning-based architectures can provide more effective feature representation for financial fraud detection. A growing body of research has begun to recognize the potential of neural networks in detecting fraudulent financial behavior. For instance, Wyrobek [16] compared the accuracy and efficiency of seven different classifiers in assessing the reliability of financial statements from 54 companies over the past 50 years. The study revealed that deep neural networks generally achieved the highest recall rate across most scenarios. Similarly, Huang [17] evaluated various machine learning models for identifying financial fraud among listed companies, finding that artificial neural networks (ANNs), support vector machines (SVMs), and random forests performed exceptionally well, each achieving accuracy rates exceeding 80%.

Despite this progress, neural-network-based fraud detection in listed-company settings remains limited, and the integration of deep feature learning with strong downstream classifiers has not yet been sufficiently explored. However, relatively few studies have explicitly combined deep feature learning with margin-based classification in the context of listed-company financial fraud detection, especially in the Chinese market [18].Accurate identification of fraudulent firms is important not only for market supervision but also for improving the efficiency of early-warning systems in securities markets. Against this background, this study develops a hybrid CNN-SVM classifier for financial fraud detection in the Chinese non-financial A-share listed-company market.

To address the challenge of accurately identifying financial fraud, this paper proposes a hybrid prediction model that combines Convolutional Neural Networks (CNNs) and Support Vector Machines (SVMs). The contributions of this paper are as follows:

1. To address class imbalance in financial fraud detection among Chinese listed companies, this study applies random oversampling to the training data so as to improve minority-class learning and support fairer model comparison.
2. The proposed framework incorporates a Convolutional Neural Network (CNN) to automatically learn feature representations from processed financial indicators, thereby improving the model’s ability to capture nonlinear patterns in fraud-related data.
3. The proposed CNN-SVM model combines CNN-based representation learning with SVM-based final classification and is evaluated against benchmark models to assess its effectiveness, efficiency, and potential value for listed-company fraud screening.

Against this background, this study develops a hybrid CNN-SVM classifier for financial fraud detection in the Chinese non-financial A-share listed-company market.The remainder of this paper is organized as follows: Section 2 presents the model methodology, outlining the specifics and recognition process of the CNN-SVM model. Section 3 discusses the data and feature processing, including data description and sources, feature selection and visualization, as well as sample preprocessing. Section 4 focuses on the selection of model evaluation criteria and presents the results of the model’s performance. Section 5 provides a comparison of the proposed model with other approaches, including statistical significance tests and further discussion. Finally, Section 6 summarizes the research findings, addresses limitations, and offers directions for future research.

## 2. Methods

**Fig 1:**
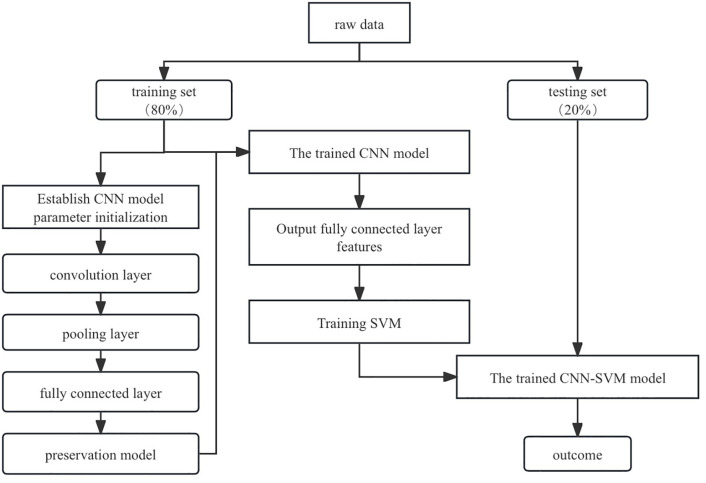
Full workflow of the proposed CNN-SVM framework for financial fraud detection.

To improve the detection accuracy of financial fraud, this paper proposes a hybrid model that combines convolutional neural networks (CNNs) with support vector machines (SVMs). The model first loads and preprocesses the data, then divides the dataset into training, validation, and test sets, applies oversampling only to the training set, and finally standardizes the features. The data are reshaped to fit the input format of a one-dimensional CNN. The CNN model consists of one convolutional layer, one pooling layer, one flattening layer, and two fully connected layers. Early stopping was used during the training process to prevent overfitting. The intermediate feature representations learned by the CNN are then transferred to the SVM for final classification. The SVM uses a linear kerneland is trained on the representations generated by the CNN, after which the same transformation is applied to the test data for classification. The steps of the model are shown in Fig 1. The details about the model are as follows.

### 2.1. CNN Parameter Selection

**Fig 2:**
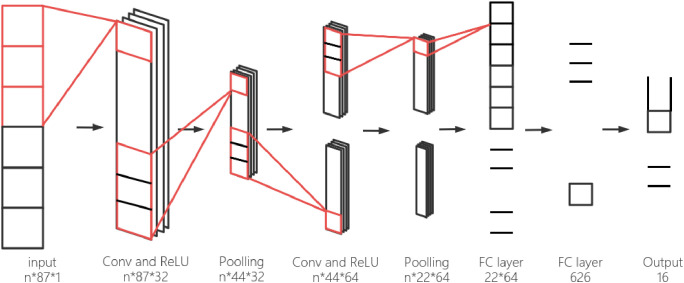
Architecture of the CNN feature extractor used in the CNN-SVM framework.

This paper employs a one-dimensional convolutional neural network (1D-CNN) to adaptively extract latent patterns associated with financial fraud in listed companies.The model structure and parameter settings are shown in Table 1 and Fig 2.

**Table 1:** Main parameter settings of the proposed CNN-SVM model, including CNN architecture and optimization choices.

parameter name	parameter value
Kernelsize of convolution kernel	3 × 3
convolution layer	1
Number of filters	32
Filter size	3
Maximum pooling layer	1
Pooling layer window size	2 × 2
fully connected layer	2
Number of fully connected layer neurons	128
Activation functions	ReLU, Sigmoid
Input data dimension	Adjust according to the number of dataset features
learning rate	Adam optimizer, which adaptively adjusts the learning rate

This CNN model uses a 3 × 3 convolutional kernel with a single convolutional layer containing 32 filters, each of size 3.These filters extract different features from the input data. Next, there is a 2 × 2 max-pooling layer, which reduces the size of the feature maps by selecting the maximum value from each 2 × 2 region, helping to improve computational efficiency and prevent overfitting.The model has two fully connected layers, each with 128 neurons, responsible for further processing the extracted features and making the final predictions. The convolutional layer uses the ReLU activation function, the output layer uses the Sigmoid activation function, and the Adam optimizer is adopted for model training.The specific parameter choices are as follows:

1. Input Layer

The data dimensions are adjusted to reshape the data into a three-dimensional array to fit the input dimensions of the CNN. The CNN proposed in this paper is one-dimensional, with a single-channel input. A total of 87 features are selected, with each company’s data for each year considered as a sample. The original dataset contains 7,429 samples, including 6,770 non-fraud samples and 659 fraud samples. After data splitting, the minority class in the training set is oversampled to improve class balance. Each sample is ultimately reshaped to the format (87, 1) before being entered into the CNN. The 1D-CNN is well suited to the input data structure. Each firm-year observation consists of 87 standardized indicators across corporate governance, accounting supervision, financial condition, and operational characteristics. Though not time-series, these inputs can be arranged as a one-dimensional feature sequence, enabling convolutional filters to capture local combinations and short-range dependencies. Compared with a conventional MLP, the convolution operation leverages local connectivity and weight sharing, reducing parameter count and improving generalization given limited fraud samples. Unlike RNN-based models designed for temporal sequences, this task involves firm-year tabular inputs rather than dynamic trajectories. Thus, 1D-CNN offers a more suitable and computationally efficient architecture for extracting latent representations from this dataset.

2. Convolutional Layer

As shown in Fig 2 the convolutional layer is the core component of a CNN. In this model, the convolutional layer uses 32 convolutional kernels, each with a size of 3. The convolutional kernels perform convolution operations on the input feature maps. During this process, all the elements are calculated through the same convolutional kernel, meaning that the weights and bias terms are shared. The process of the convolution operation is shown in the following formula(1) [18]:

**Fig 3:**
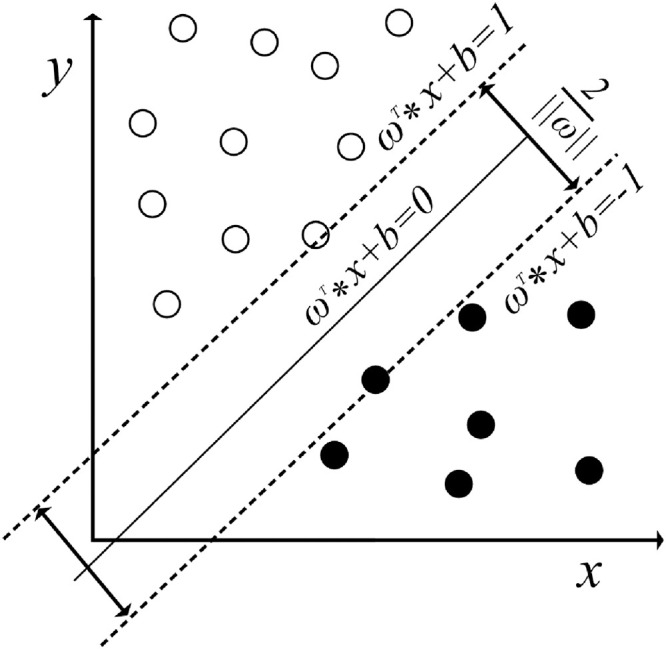
Schematic illustration of the support vector machine classification principle.


$$\begin{array}{c} \overset{r}{\underset{j}{x}}=f\left(\sum \overset{r-\text{1}}{\underset{i}{x}}\times \overset{r}{\underset{i,j}{k}}+\overset{r}{\underset{j}{b}}\right) \end{array}$$ (1)

where $\overset{r}{\underset{j}{x}}$ denotes the jth output feature map obtained by the convolution operation of the rth layer, $\overset{\text{r}}{\underset{\text{j}}{\text{b}}}$ represents the bias of the jth convolution kernel of ther th layer network, $\overset{r}{\underset{i,j}{k}}$ represents the j th convolution kernel of the rth layer and the ith input feature map operation, and f is a nonlinear activation function.

3. Activation function

The convolutional and hidden dense layers use the ReLU activation function, while the output layer uses the sigmoid activation function. ReLU is adopted because it supports efficient nonlinear feature learning in the hidden layers, whereas sigmoid is appropriate for the final binary output because it maps the prediction to a probability in the range of 0–1. Their roles in the model are illustrated in Fig 2. Its expression is shown in Formula (2) [18], which represents the data obtained after the convolution operation.


$$\begin{array}{c} R_{eLU}(x)\left\{ \begin{array}{c} x,x>\text{0}\\ \text{0},x\leq \text{0} \end{array}\right. \end{array}$$ (2)

4. Pooling Layer

A max-pooling layer with a pool size of 2 is applied after convolution to down-sample the learned feature maps. This operation reduces dimensionality and computational cost while preserving the strongest local responses.The calculation formula of the pooling operation is shown in Formula (3) [18]:


$$\begin{array}{c} p(i,j)=\frac{\text{1}}{\omega^{\text{2}}}\sum_{u=(i-\text{1})\omega +\text{1}}^{i\omega }\sum_{v=(j-\text{1})\omega +\text{1}}^{j\omega }a(u,v) \end{array}$$ (3)

where a(u,v) represents the values of rows and columns in the input matrix of the pooling layer, p(i,j) represents the value of the ith row j of the pooling layer output matrix, and ω represents the boundary value of the participating set region.

5. Flattening Layer and Fully Connected Layer

After convolution and pooling, the multidimensional feature maps are flattened into a one-dimensional vector and fed into the fully connected layers. The first dense layer with 128 neurons further integrates the extracted local patterns, and the final dense layer with one neuron produces the binary classification output. In this study, the output layer uses a sigmoid function to generate the predicted probability of financial fraud. Its model can be expressed as Formula (4) [18]:


$$\begin{array}{c} y=\omega x+b \end{array}$$ (4)

where x is the input to the fully connected layer, ω is the weight matrix, and b is the bias vector.

### 2.2. SVM feature extraction

The support vector machine (SVM) is a machine learning algorithm proposed by Cortes C et al. [19] and is based on the VC dimension and the principle of structural risk minimization in statistical learning theory. The core objective of SVM is to determine a hyperplane that optimally separates data of different categories while maximizing the margin. The hyperplane that maximizes the distance between the samples of two classes is called the separating hyperplane, and the geometric definition of the separating hyperplane can be mathematically expressed as (5) [20]:


$$\begin{array}{c} \omega^{T}\cdot x+b=\text{0} \end{array}$$ (5)

where ω represents the normal vector of the optimal hyperplane and b is the distance between the hyperplane and the origin.

If the hyperplane in the feature space is denoted as (ω, b), then in order for this plane to correctly classify the sample points, it should satisfy:


$$\begin{array}{c} y_{i}(\omega^{T}\cdot x+b)\geq \text{1} \end{array}$$ (6)

That is, for any sample point $x_{i}$ on one side, it should satisfy that when the projection of the point on the normal vector is greater than the threshold c, if the sample is predicted as a positive sample, then $y_{i}=\pm \text{1}$, where the value of c should satisfy $\omega^{T}>c$.As shown in Fig 3, according to the definition of support vectors, the ultimate classification goal of the SVM is as follows:


$$\begin{array}{c} \underset{\omega ,b}{min}a\left\|\omega \right\| \end{array}$$

$$\begin{array}{c} s.t.y_{i}\left(\omega^{T}*x+b\right)\geq \text{1},i=\text{1},\text{2},\ldots ,m \end{array}$$ (7)

To simplify the computation, it can be transformed into a dual problem for the solution. Using the method of Lagrange multipliers, the above equation can be formulated as:


$$\begin{array}{c} L(\omega ,b,a)=\frac{\text{1}}{\text{2}}\left\|\omega^{\text{2}}\right\|+\sum_{i=\text{0}}^{n}a_{i}(y_{i}(\omega^{T}*x+b)) \end{array}$$ (8)

By taking the partial derivatives of the aforementioned equation and setting them to 0, and then applying the Karush–Kuhn–Tucker (KKT) conditions, the values of $\overset{*}{\underset{i}{a}}$, $\overset{*}{\underset{i}{b}}$, and $\overset{*}{\underset{i}{\omega }}$ can be sequentially determined.

### 2.3. Model implementation steps

1. Data Loading and Preprocessing

The first step in the process involves importing the necessary libraries and loading the dataset. Once the data is loaded, it undergoes a cleaning process where irrelevant columns, such as id and year, are removed. These columns do not contribute significantly to the predictive power of the model. The target variable for fraud detection is Financialfraud, which is a binary variable indicating fraud (1) or non-fraud (0).

Next, the class distribution of the target variable is examined to assess the imbalance between fraud and non-fraud cases. Because fraudulent instances are substantially underrepresented, random oversampling is applied only to the minority class in the training set so that the classifier can learn fraud-related patterns more effectively without altering the natural class distribution of the validation and test sets.

2. Data Splitting and Standardization

The dataset is then divided into features (X) and the target variable (y), after which a stratified split is used to construct the training, validation, and test sets while preserving the original class distribution in the evaluation subsets.In the experiments, the dataset was divided into training, validation, and test sets at a ratio of 60:20:20. To avoid information leakage, random oversampling was applied only to the training set, while the validation and test sets retained the original class distribution.

To ensure optimal model performance, the features are standardized. Standardization is crucial as it scales the features such that each has a mean of zero and a standard deviation of one. This process is particularly important for machine learning models, including Support Vector Machines (SVMs), that are sensitive to feature scale.

3. Reshaping Data for CNN Input

Convolutional Neural Networks (CNNs) typically require three-dimensional input data with the shape (samples, time-steps, features). Although the data in this case does not represent time-series information, it is reshaped to fit the requirements of the CNN model. Each sample is transformed into a 3D tensor, where the second dimension corresponds to the number of features, and a single channel is used for each sample. This reshaping allows the CNN to process the features as if they were part of a sequential pattern, even though they are not inherently sequential.

4. Building the CNN Model

The model is built using a Sequential architecture from Keras, incorporating several key layers. The first layer is a 1D convolutional layer (Conv1D), which learns 32 different feature maps from the input data. The kernel size is set to 3, meaning each filter will examine 3 consecutive values at a time. The ReLU activation function is applied to introduce non-linearity into the model, and the input shape is defined based on the number of features in each sample.

Following the convolutional layer, a max-pooling layer (MaxPooling1D) is applied to reduce the dimensionality of the feature maps. This layer takes the maximum value over every two consecutive values in the feature maps, thereby down-sampling the data and reducing computational complexity.

The output of the pooling layer is then flattened using the Flatten layer, converting the 2D feature maps into a 1D vector. This vector is then fed into fully connected (dense) layers. The first dense layer contains 128 neurons, and the ReLU activation function is again used. The final dense layer has a single neuron with a sigmoid activation function, which outputs a probability between 0 and 1, indicating the likelihood of fraud (1) or non-fraud (0).

The model is compiled using the Adam optimizer, binary cross-entropy as the loss function, and accuracy as the evaluation metric.

5. Training the CNN Model

The CNN model is trained on the reshaped data, with 20 epochs and a batch size of 64. To prevent overfitting, early stopping is implemented, which halts training if the validation loss does not improve for three consecutive epochs. This ensures that the model does not continue to learn noise from the data. During training, both the training and validation loss are monitored, and the training progress is visualized by plotting these values over the epochs.

6. SVM on CNN Output

After training the CNN model, the learned feature representations are transferred to a Support Vector Machine (SVM) classifier to further improve final classification performance. These CNN-derived representations are used as inputs to the SVM so as to refine the decision boundary in the fraud-detection task. The SVM classifier is trained using a linear kernel. This choice is based on the consideration that the CNN has already captured the major nonlinear structure in the original financial indicators, so the subsequent classification task can be effectively completed by a maximum-margin linear separator in the learned representation space. Compared with nonlinear kernels, the linear kernel also offers lower computational burden, stronger stability, and easier model deployment. This hybrid approach therefore leverages the CNN for nonlinear feature extraction and the linear SVM for efficient and robust final classification.

7. Model Evaluation

Finally, the performance of the hybrid CNN-SVM model is evaluated using several classification metrics. A confusion matrix is generated to assess the model’s ability to distinguish between fraud and non-fraud cases. Additionally, a classification report provides further insight into the model’s precision, recall, F1-score, and overall accuracy. These metrics are particularly important for imbalanced classification tasks, where the focus is often on ensuring that the minority class (fraud cases) is adequately identified.

In summary, the proposed CNN-SVM model combines the representation-learning capability of CNNs with the margin-based classification strength of SVMs. The CNN is used to learn informative feature patterns from structured financial indicators, whereas the SVM serves as the final classifier for fraud identification. This hybrid design is intended to improve classification performance on complex and imbalanced financial fraud data while maintaining a practically feasible inference cost.

## 3. Data

### 3.1. Data declaration and sources

#### 3.1.1. Data statement.

In the Chinese market, the behavior of publicly listed companies in disclosing corporate information strictly adheres to a legal framework centered on the Securities Law of the People’s Republic of China, supplemented by the Measures for the Administration of Information Disclosure by Listed Companies and relevant interpretations of the Accounting Standards for Enterprises. These legal provisions and policies collectively establish a comprehensive information disclosure framework that guides listed companies in the thorough, timely, and accurately release various important information, including financial reports, significant events, related-party transactions, and changes in shareholding.

Moreover, in compliance with the Personal Information Protection Law and data security and privacy protection policies, listed companies ensure that no personal data are disclosed when releasing information, thereby safeguarding data security and protecting investors’ rights. Consequently, we acquire this publicly available information in a reasonable and lawful manner, with no involvement of personal privacy issues and no risk of data leakage. All the data and code can be publicly accessed in compliance with relevant regulations without involving third-party ownership issues. All data and code are publicly accessible, and the links to access them are provided in the supporting information section.

The fraud samples in this study are all derived from publicly disclosed penalty cases by regulatory authorities, ensuring the reliability of the labels. Although there may be undiscovered fraudulent behaviors, their characteristics might differ from known fraud patterns. In the future, the model’s adaptability can be enhanced by continuously updating the dataset to include new cases.We also acknowledge that the fraud labels are based on publicly disclosed penalized firms, which may not fully capture undetected or unpenalized fraudulent behavior. Therefore, the model should be interpreted as learning patterns from confirmed fraud cases, and this potential sample-selection bias should be considered when evaluating the generalizability of the results.

This study uses publicly available firm-level data and does not involve human participants or personal information.From an ethical and practical perspective, false-positive predictions may incorrectly identify legitimate firms as high-risk, which could result in reputational harm, unnecessary regulatory attention, or distorted investment decisions. Therefore, the proposed model should be used as an early-warning support tool rather than as a standalone basis for punitive or public labeling decisions.

#### 3.1.2. Variable selection.

In this paper, A-share listed companies from 2007–2022 were taken as the initial research sample, and the data were obtained from the CSMAR database and Wind database. This paper uses Excel and Stata to process the raw data as follows: (1) Exclusion of Financial companies. Due to the significant differences between the financial structure and operating characteristics of financial companies and nonfinancial companies, to ensure the accuracy of the overall data analysis, we excluded the sample of financial companies. (2) Handling missing values. Empty values, empty spaces, and the string “None,” which represents the missing characters in the sample, were identified and removed. After this processing, we obtained a total of 7429 sample data points, covering 90 industries.

The label of this article is financial fraud (financial fraud), which refers to the research of CHEN Yan et al. [21] research, screening 2007–2022. They screened cases from 2007 to 2022 identifying instances of financial fraud (inflated profits, false assets records, misleading statements, information material omissions, and disclosure violations) published by the China Securities Regulatory Commission and its local branches, the Ministry of Finance, and the Shenzhen, and Shanghai Stock Exchange punishment of nonfinancial A-share listed companies as a training sample. For these listed companies, a binary variable was set to indicate whether financial fraud occurred in a given year.Each fraud case was matched to the corresponding firm-year observation according to the reporting year identified in the regulatory penalty disclosure. If a case involved multiple reporting years, each affected firm-year was labeled separately. This matching strategy ensures that the fraud label is aligned with the annual financial data used for model training. If the Company, the value is 1; if not, the value is 0. This paper selects data from 659 nonfinancial A-share listed companies punished by the China Securities Regulatory Commission (CSRC) and its local branches, the Ministry of Finance, and the Shenzhen and Shanghai Stock Exchanges for financial fraud between 2007 and 2022. Table 2 shows the distribution of the years of financial fraud. According to the Table 2, the number of financial frauds peaked in 2014 and 2015, accounting for 14.16% and 19.3% of the total sample, respectively.To ensure data integrity, the firm-level records from the CSMAR and Wind databases were cross-checked by firm identifier, reporting year, and key financial fields. Duplicate observations, invalid entries, and unresolved missing values were removed during preprocessing to ensure consistency between the fraud labels and the corresponding feature records.

**Table 2:** Year-wise distribution of confirmed financial fraud cases in the sample.

a particular year	Number of financial fraud cases	proportion
2007	12	1.8%
2008	21	3.2%
2009	25	3.8%
2010	27	4.1%
2011	41	6.2%
2012	91	13.8%
2013	89	13.5%
2014	96	14.6%
2015	127	19.3%
2016	24	3.6%
2017	19	2.9%
2018	24	3.6%
2019	27	4.1%
2020	21	3.2%
2021	14	2.1%
2022	1	0.2%
amount to	659	100%

### 3.2. Features

#### 3.2.1. Feature selection.

This article references the practices of Weihua Zhou et al. [7] who select characteristic variables from four aspects: corporate governance, accounting supervision, financial indicators, and enterprise operation. First, corporate governance is a key indicator for measuring the efficiency of corporate management and the perfection of the internal balance mechanism. Well-managed companies are often able to significantly reduce the occurrence of financial fraud [22]. The evaluation of corporate governance in this paper focuses on core first-level indicators such as the structure of the board of directors, the size of the board of independent director board, and whether roles are combined (e.g., CEO and chairperson). It also looks at shareholders’ equity (such as the shareholding ratio of major shareholders, equity concentration, etc.) and management characteristics (such as management experience, educational background, etc.). These primary indicators are refined into two-level indicators, such as the size of the board of directors and the proportion of independent directors, jointly building a framework to comprehensively measure the level of corporate governance to reveal the degree of perfection and potential risks of corporate governance [23]. Second, accounting supervision plays a pivotal role in preventing and combating of financial fraud. By formulating and implementing strict accounting standards and audit standards, accounting supervision institutions ensure the standardization and accuracy of corporate financial reports to reduce the likelihood of financial fraud [24]. This paper focuses on audit quality, the transparency of information disclosure, and the selection of audit institutions, which are the key links of accounting supervision. Audits by Big Four accounting firms usually represent high quality and independence, which enhances the credibility of financial information. The “unqualified opinion” audit report reflects the true financial situation and reduces the risk of information asymmetry. The rationality of the audit cost guarantees the audit quality, and too low of a cost may affect the audit procedure [25]. In addition, the company’s asset and liability status should be investigated to assess its financial robustness, and a strong financial position lowers the motivation for fraud. These indicators reflect the effectiveness of accounting supervision and offer critical insights for fraud prevention. Third, financial indicators are a direct embodiment of the financial situation and development potential of enterprises, serving as key tools in detecting financial fraud [26]. In cases of financial fraud, companies often manipulate financial indicators to hide the real situation or enhance their performance. In this work, the financial indicators of multiple dimensions, such as profitability, solvency, operational efficiency, asset structure, and growth ability, are selected as the first-level indicators and further refined into second-level indicators, such as the net profit margin of total assets, return on net asset assets and the asset‒liability ratio [27]. These indicators together form a comprehensive financial analysis system that helps assess the financial health and development potential of enterprises, providing investors and regulators with accurate information to detect and prevent fraud risks. Fourth, financial fraud is often concealed within abnormal behavior or management loopholes of enterprise operations; therefore, this paper adds indicators of the enterprise operation level to better identify the financial fraud of enterprises [28]. This paper focuses on market valuation, investment and cash flow management, shareholder and management relationships, operating costs, regional and industry characteristics, and basic information about the company. Under these first-level indicators, this paper further refines the construction of second-level indicators such as the cash proportion of fixed asset payments and bank shareholding, thus constructing a detailed and comprehensive analysis framework. This detailed framework helps better assess the operational status of enterprises and identify potential financial fraud risk [29].S1 Table presents the statistics of the characteristic values of the fraudulent samples (excerpts), and S2 Table presents the statistics of the characteristic values of the non-fraudulent samples (excerpts), with the complete indicators provided in the Supplementary Materials (S1 Fig).

#### 3.2.2. Feature engineering.

**Fig 4:**
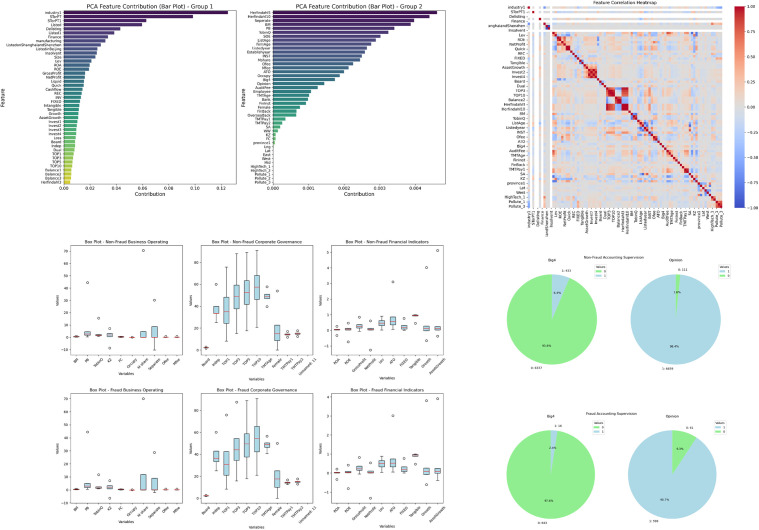
Visualization features recognition (a) PCA chart; (b) Heatmap of the positive correlation between audit quality and information disclosure transparency; (c) Boxplot of non-fraud and fraud samples; (d)Pie chart of the distribution of audit quality and audit opinions.

Although convolutional neural networks (CNNs) are effective at extracting key features, the contributions of these features remain somewhat of a black box. To clarify the contributions of these features, this study employs principal component analysis (PCA), correlation analysis, and box plots to analyze the feature variables across four key dimensions systematically: corporate governance, financial oversight, financial indicators, and corporate operations. In addition, the PCA results provide an interpretable indication of the relative importance of variables through their contributions to the principal components with higher explained variance. This complements the four-category feature framework and helps highlight the indicators that are more strongly associated with fraud identification.The results are illustrated in Fig 4.

First, the PCA results indicate that the proportion of independent directors and the size of the board strongly contribute to the corporate governance dimension, significantly influencing the overall level of governance. Additionally, variables related to financial indicators, such as gross profit, net profit, return on assets (ROA), and return on equity (ROE), also hold significant weight in the PCA, underscoring the central roles of profitability and asset management efficiency in assessing a company’s financial health and growth potential.

Second, the correlation analysis reveals the interdependencies among the variables. For example, in the financial oversight dimension, the positive correlation between audit quality and the transparency of information disclosure suggests that higher audit quality is often associated with more transparent disclosures, which is crucial for preventing financial fraud. Furthermore, the positive correlation between profitability and solvency in the analysis of financial indicators suggests a close relationship between a company’s profitability and its ability to meet debt obligations, which is vital for assessing the financial health of the organization. In terms of corporate operations, the positive correlation between investment decisions and cash flow management underscores the importance of effective cash flow management in supporting corporate investment decisions.

Finally, the analysis of box plots for nonfraudulent and fraudulent samples reveals that the fraudulent samples present greater volatility and outliers in terms of corporate governance and operational metrics, while nonfraudulent samples present a more stable and concentrated distribution. This finding indicates that deficiencies in corporate governance structures and instability in corporate operations may be significantly correlated with financial fraud.The pie chart illustrates the distribution of the audit quality and the audit opinions metrics within the financial supervision. Analysis of the chart reveals that the proportion of audits performed by the Big Four accounting firms in fraud cases is comparatively low, while the proportion of unqualified opinion reports is notably high. This suggests a positive correlation between high-quality audits and a reduction in the risk of financial fraud.

In summary, this paper provides a comprehensive analysis of the distribution of feature values between fraudulent and nonfraudulent samples, identifying potential risk indicators associated with financial fraud. Shortcomings in corporate governance, weak financial oversight, abnormal fluctuations in financial indicators, and instability in corporate operations may all serve as indicators of financial misconduct. Additionally, the results emphasize the critical role of high-quality audit supervision in mitigating the risk of financial fraud. By analyzing the contributions of feature variables, we can effectively reduce data dimensionality, decrease computational complexity, and eliminate redundant features, thereby improving both the training speed and the accuracy of the model.

### 3.3. Data preprocessing

#### 3.3.1. Random oversampling processing.

**Fig 5:**
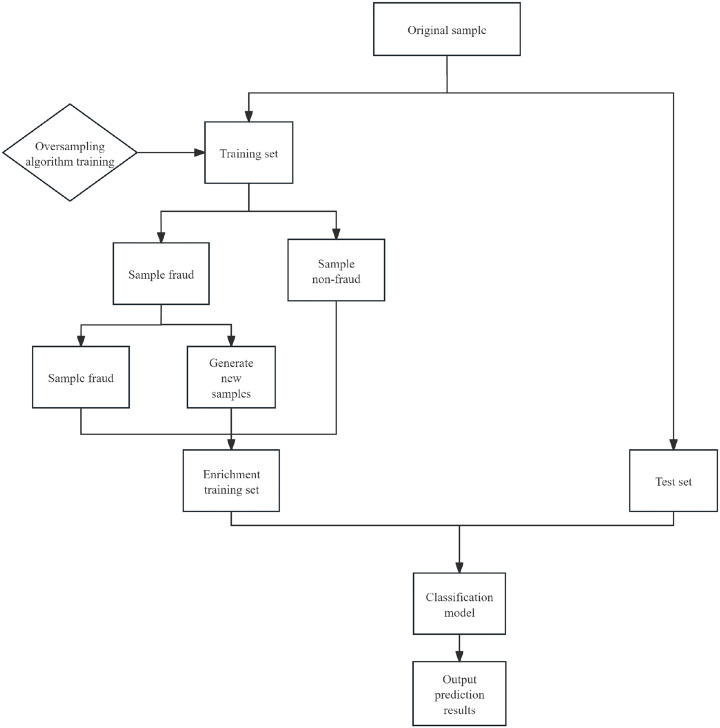
Workflow of the random oversampling procedure.

When the number of samples in one category far exceeds that in another, an unbalanced dataset is formed [30]. In this paper, using the financial fraud dataset as an example, the number of public case samples is unequal, leading to the neglect of minority samples during model training, which subsequently affects its recognition ability and reduces its accuracy. To address this issue, random oversampling is applied to balance the data. The basic principle of this algorithm lies in expanding the data by adding a sample set E to the original dataset S. The data are then expanded by adding sample collection E to the original dataset S. As a result, the size of the expanded dataset becomes the sum of the original majority class sample set $S_{maj}$, the original minority class sample set $S_{min}$, and the additional samples E, i.e., $|S|=|S_{maj}|+|S_{min}|+|E|$. Specifically, the composition of the set E is obtained by randomly selecting the samples from and replicating the minority class sample set $S_{min}$*.* This process aims to expand the original dataset S [31] and increase the total number of samples in the minority class sample set $S_{min}$ by |E|. In this way, the class distribution of the dataset S is equalized and adjusted [32].The processing flow is illustrated in Fig 5.

The treatment results are shown in Table 3, where the number of samples in the category “occurring financial fraud” increased significantly, from 659 to 6670, matching the number of samples in the “no financial fraud” category. This treatment ensures that the classifier can more accurately identify underrepresented categories and reduce costly false-negatives, thereby improving the accuracy of machine learning models in identifying the “financial fraud occurs” category.

**Table 3:** Class distribution before and after random oversampling.

	No financial fraud has occurred	Financial fraud occurs
Number of original samples	6670	659
scale	91.13%	8.87%
After oversampling		
sample size	6670	6670
scale	50.00%	50.00%

#### 3.3.2. Standardized processing.

Given the significant magnitude difference in the dataset, with capital usually in the hundreds of millions of dollars, the profit margin is generally less than 1%. To ensure that the weight of each index can be balanced in the data analysis, this paper adopts a standardized method and uses the systematic processing of the data. The specific calculation process is as follows:Z−score.

For the original variable sequence, the data is processed using the following operation formula: $X=\{x_{\text{1}},x_{\text{2}},\ldots ,x_{n}\}$


$$\begin{array}{c} y_{i}=\frac{x_{i}-x_{mean}}{std} \end{array}$$ (9)

Where $x_{mean}$ represents the mean of the original variable sequence X, and std is its standard deviation. With the above calculation, we finally obtain the standardized variable sequence.$Y=\{y_{\text{1}},y_{\text{2}},\ldots ,y_{n}\}$

## 4. Results

### 4.1. Model evaluation

The identification of financial fraud in listed companies is essentially a binary classification problem. Once classification is conducted, the accuracy of the detection method must be evaluated. The confusion matrix is an important tool for measuring the effectiveness of methods, which visualizes the performance of classification algorithms and helps identify cases of misclassification and correct classification [32]. The confusion matrix categorizes all detected users into four classes on the basis of the actual classification and detection results: true positive (TP), false-negative (FN), false-positive (FP), and true negative (TN). TP and TN represent the accurately classified portions by the model, and the higher their proportion is, the more effective the detection. Table 4 shows the confusion matrix for identifying occurrence of financial fraud.

**Table 4:** Structure of the confusion matrix used for financial fraud detection.

	Detection of financial fraud	bnormal detection
Actual occurrence of financial fraud	TP(True Positive)	FN(False-Negative)
Actual Normal	FP(False-Positive)	TN(True Negative)

The area under the curve (AUC) is commonly used as a metric for evaluating the performance of machine learning binary classification problems [33]. Specifically, in a binary classification task, the area under the curve (AUC) measures the classifier’s ability to rank positive samples ahead of negative samples. When the AUC approaches 1, it indicates better performance of the classifier in arranging positive and negative instances, meaning that it is more likely to correctly rank positive samples ahead of negative samples. Conversely, when the AUC approaches 0, it indicates poorer performance of the classifier, possibly resulting in more frequent rankings of negative samples ahead of positive samples.

For a practical classifier, the range of AUC values is typically between 0.5 and 1. Assuming a set of samples where the number of positive samples is N and the number of negative samples is M, there are a total of N*M binary sample combinations. For each combination, the classifier’s confidence and sort these confidence values can be computed and sorted. The calculation formula for the AUC is shown in Equation (8), where the APS (all positive samples) represents all positive samples.


$$\begin{array}{c} AUC=\frac{\sum_{\text{APS }}\;\;\text{rank}-\frac{M(M+\text{1})}{\text{2}}}{M*N} \end{array}$$ (10)

The accuracy (overall classification accuracy) is a metric for evaluating the performance of a classification model. It represents the ratio of correctly classified samples to the total number of samples [34]. Accuracy is very useful when the class distribution is relatively balanced and the costs of false positives (FP) and false negatives (FN) are similar [35]. However, when the dataset is imbalanced, accuracy can be misleading [36]. The calculation formula for the Accuracy is shown in Equation (11).


$$\begin{array}{c} accuracy=\frac{\left(TP+TN\right)}{TP+FP+TN+FN} \end{array}$$ (11)

Precision refers to the proportion of samples identified as positive by the model that are actually positive among all samples identified as positive [32]. A higher value indicates that the model is more accurate in identifying fraudulent behavior. Precision is defined as the ratio of correctly predicted positive samples to the total number of samples that were predicted as positive. Please refer to Formulas(12) for the calculation of this metric [37].


$$\begin{array}{c} precision=\frac{TP}{TP+FP} \end{array}$$ (12)

Recall, also known as the true positive rate, refers to the proportion of all actual positives that the model successfully identifies as positive[42. A higher value indicates that the model has a stronger ability to detect real instances of fraudulent behavior. Recall is defined as the ratio of correctly predicted positive samples to the total number of actual positive samples. The calculation formula for the Accuracy is shown in Equation (13).


$$\begin{array}{c} ecall=\frac{TP}{TP+FN} \end{array}$$ (13)

The F1 score is a metric that combines precision and recall to evaluate the model’s performance [38,39]. It is the harmonic mean of precision and recall, balancing both metrics. In the F1 score, precision measures the accuracy of the model, whereas recall measures the completeness of the model. A higher value indicates that the model has a stronger ability to balance precision and recall.F1 score is the harmonic mean of precision and recall, where beta is typically set to 0.5, 1, or 2 to represent the weighting of precision. The calculation formula for the Accuracy is shown in Equation (14).


$$\begin{array}{c} F\text{1}=\left(\text{1}+\beta^{\text{2}}\right)\frac{precision*recall}{\beta^{\text{2}}precision+recall} \end{array}$$ (14)

### 4.2. Experimental verification

To validate the effectiveness and accuracy of the proposed algorithm, experiments were conducted on an experimental platform equipped with an Intel Core i9-13900H CPU@2.6 GHz, featuring 64 bits, 14 cores, and 20 threads. TensorFlow and Keras, two deep learning frameworks, were utilized for implementation. The implementation was based primarily on TensorFlow/Keras for the CNN component and Scikit-learn for data preprocessing, oversampling, and the SVM/LR classifiers. To improve reproducibility, randomization was controlled with a fixed random seed (random_state = 42) in data splitting and oversampling.The experimental data were sourced from financial datasets publicly available from listed companies between 2007 and 2022; for detailed information, refer to Section 2 of this paper.This study compared four models: support vector machine (SVM), logistic regression (LR), CNN + SVM (CNN for feature extraction with SVM for classification, abbreviated as CNN-SVM), and CNN + LR (CNN for feature extraction with LR for classification, abbreviated as CNN-LR).

All models were trained using the same data-splitting strategy. The original dataset was first divided into training, validation, and test sets, after which random oversampling was applied only to the training set. Specifically, 60% of the data were used for training, 20% for validation, and the remaining 20% for testing.

Table 5 reports the main classification results for the core benchmark models, while the extended comparison in the subsequent bootstrap analysis further includes RF and XGBoost to provide a broader baseline assessment.According to the results in Table 5, the SVM model performed relatively poorly in terms of accuracy, recall, precision, and F1 score, with values of 0.55, 0.66, 0.54, and 0.59, respectively. The logistic regression (LR) model slightly outperformed the SVM model in all metrics but still exhibited mediocre performance. In contrast, the models based on CNNs (CNN-SVM and CNN-LR) significantly outperformed the traditional models across all the metrics. In particular, CNN-SVM achieved high scores in all the metrics, with accuracies, recalls, precisions, and F1 scores of 0.97, 0.99, 0.96, and 0.97, respectively. The performance of CNN-LR was also quite impressive, slightly lower than that of CNN-SVM but still far superior to the traditional models, with accuracies, recalls, precisions, and F1 scores of 0.95, 0.97, 0.93, and 0.95, respectively. Thus, it can be concluded that the proposed CNN-SVM model in this paper outperforms the traditional SVM and LR models in this task.

**Table 5:** Test-set performance comparison of the fraud detection models.

Model	Evaluation indicators			
	${𝐀}_{UC}$	${𝐑}_{ecall}$	${𝐏}_{recision}$	${𝐅}_{1}$
SVM	0.55	0.66	0.54	0.59
LR	0.61	0.61	0.61	0.61
CNN-SVM	0.97	0.99	0.96	0.97
CNN-LR	0.95	0.97	0.93	0.95

**Fig 6:**
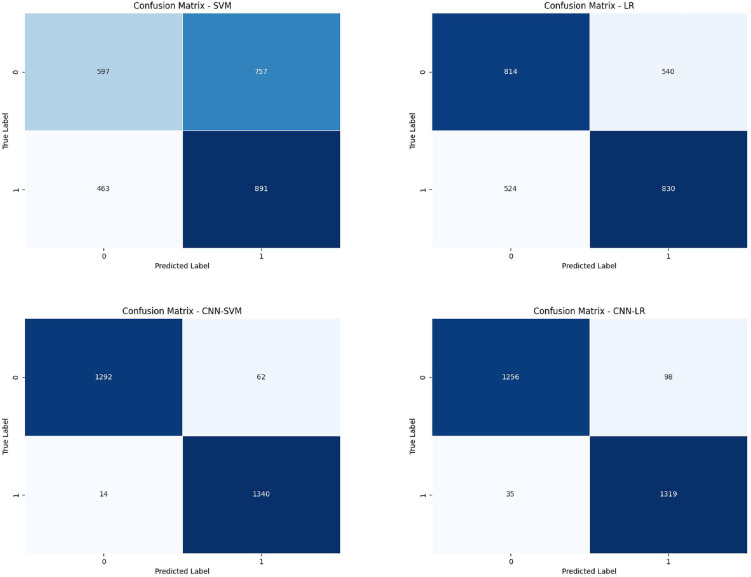
Confusion matrices of the four benchmark models on the test set.

The confusion matrix is used to evaluate the performance of classification models by illustrating correct classifications and error types..Fig 6 presents the confusion matrices of four models: SVM, Logistic Regression (LR), Convolutional Neural Network with Support Vector Machine (CNN-SVM), and Convolutional Neural Network with Logistic Regression (CNN-LR). The x-axis of each matrix represents the predicted labels, while the y-axis represents the actual labels, with two categories: 0 and 1. Darker colors indicate higher quantities. By comparing these matrices, we can observe the performance differences among different models. The confusion matrices reveal that CNN-SVM and CNN-LR perform better in reducing false-negatives, demonstrating their ability to more accurately identify positive instances.

The receiver operating characteristic (ROC) curve is a chart used to evaluate the performance of classification models. For clarity, the confusion matrices are presented with actual labels on the y-axis and predicted labels on the x-axis, where 0 denotes non-fraud and 1 denotes fraud.It demonstrates the model’s ability to discriminate by comparing the true positive rate (TPR) and the false-positive rate (FPR). The FPR, represented on the horizontal axis as FalsePositiveRate=FP/(FP+TN), indicates the percentage of negative samples incorrectly classified as positive among all actual negative samples. The TPR, represented on the vertical axis as TruePositiveRate=TP/(TP+FN), indicates the percentage of positive samples correctly classified as positive among all actual positive samples^1^. Generally, the smaller the horizontal axis value and the larger the vertical axis value are, the larger the area under the ROC curve (AUC), indicating better model performance.

**Fig 7:**
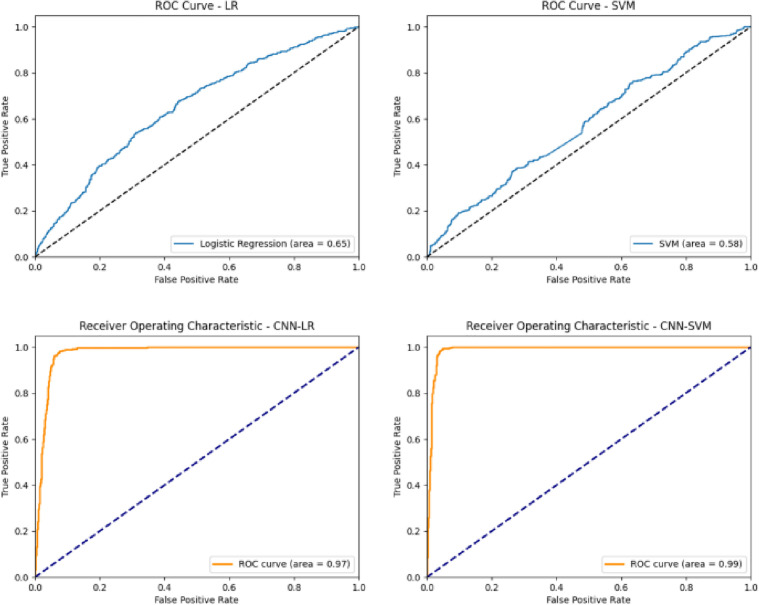
ROC curves of the four benchmark models on the test set.

Fig 7 shows the ROC curves for four models: LR, SVM, CNN-SVM, and CNN-LR. In the ROC plots, the x-axis represents the false-positive rate and the y-axis represents the true-positive rate, and each curve is labeled by model name to facilitate visual comparison.The LR model performs better than random guessing (the blue dashed line) but remains inferior to the CNN-based models. The SVM model performs the closest to random guessing, indicating poor classification performance. The CNN-LR model achieves an AUC of 0.97, indicating excellent performance that is significantly better than random guessing and shows strong classification capabilities. The CNN-SVM model achieves an AUC of 0.97, which is the best among the four models and indicates excellent classification performance. The ROC curves demonstrate that models incorporating CNNs (CNN-LR and CNN-SVM) perform exceptionally well in classification tasks.

The loss function (LOSS) is used to measure the difference between the predicted values and the actual values [40], serving as a key metric for optimizing machine learning models. The decreasing trend of the loss function indicates that the model is learning from the data and improving its predictive accuracy. The gap between training loss and validation loss can reflect whether the model is overfitting or underfitting. Ideally, we want both to be low and close to each other, indicating good performance on both the training and validation sets.

**Fig 8:**
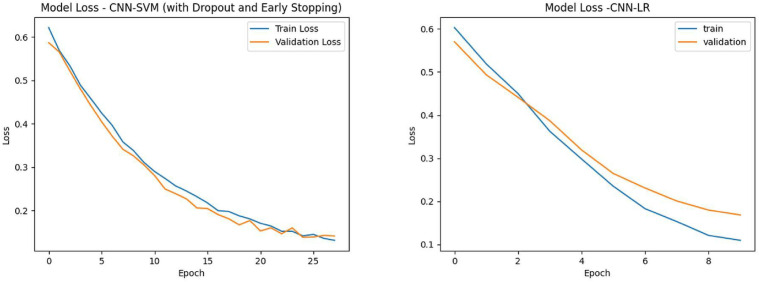
Training and validation loss curves of the CNN-SVM and CNN-LR models.

Fig 8 shows the loss curves of the CNN-SVM and CNN-LR models. For the CNN-SVM model, both the training loss and validation loss decrease as the number of epochs increases and gradually stabilize within the training process, indicating that the model has learned useful feature patterns and achieved a reasonable level of generalization. The training loss (blue line) and validation loss (orange line) of the CNN-LR model start to decrease after 8 epochs, indicating gradual improvement in its predictive performance during the learning process. A comparison reveals that the loss function of the CNN-SVM model demonstrates better stability and generalizability.

To evaluate the real-time capability of the CNN-SVM model in practical deployment, this study further tested the inference time per sample. The experiment was conducted on the same Intel Core i9-13900H CPU platform, with the test set containing 2,708 samples (20% of the total data), and the measurement was repeated 100 times to obtain the average. The results are shown in Table 6.

**Table 6:** Average single-sample inference time of different models.

Model	Average single-sample inference time(ms)	Standard deviation(ms)
CNN-SVM	1.24	0.08
CNN-LR	1.18	0.07
SVM	0.32	0.03
XGBoost	0.41	0.0.4

The CNN-SVM’s inference time per sample is 1.24 milliseconds, which is slightly higher than traditional models (SVM/XGBoost), but still within the acceptable range for financial fraud detection (usually required to be < 10ms).The time overhead primarily comes from the CNN feature extraction stage (accounting for 82%), while the SVM classification only accounts for 18%, which aligns with the model design expectations. Although the inference time of CNN-SVM is 3.8 times longer than that of SVM, the significant improvement in AUC (0.97 vs 0.55) and recall rate (0.99 vs 0.53) (see Table 5) demonstrates that the slight time cost is compensated by a qualitative leap in detection performance. This trade-off holds practical value in the financial risk control field, as the cost of missing fraud cases is much higher than the computational resource overhead.

### 4.3. Performance testing

To further validate the superiority of the proposed model, this study also includes Random Forest (RF) and XGBoost as additional benchmark models and compares their performance with that of Logistic Regression (LR) and Support Vector Machine (SVM) [41]. Each of the five models was trained 1000 times, and the precision, recall, F1 score, and accuracy metrics from each training run were saved for comparative analysis. Further analysis is provided below in terms of statistical testing, analysis of results and significance.

#### 4.3.1. Descriptive statistics.

**Fig 9:**
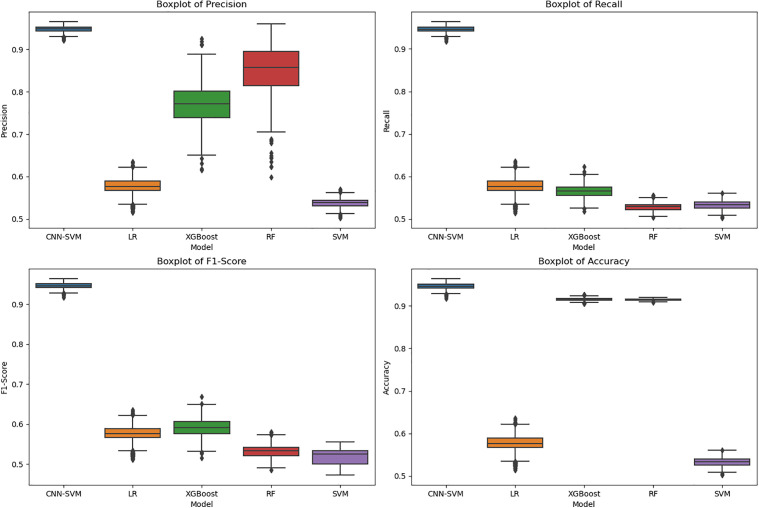
Boxplots of performance-metric distributions across the compared models.

Table 7 presents the descriptive statistics of the obtained metric values, while Fig 9 shows a box plot based on the data distribution. The CNN-SVM model demonstrates impressive performance in identifying fraudulent cases. It achieves a high mean precision of 0.947974, with minimal standard deviation (0.006803), indicating its consistency in making reliable predictions. The precision ranges from 0.920581 to 0.965054, further confirming the model’s robust ability to distinguish fraud cases. In terms of recall, the model performs similarly well, with a mean recall of 0.946045 and a small standard deviation (0.006942), highlighting its effectiveness in detecting rare instances of fraud. The recall values range from 0.917651 to 0.963811, reinforcing the model’s ability to capture fraud cases with high accuracy. With an F1-score of 0.945986 and low variation, the model strikes a strong balance between precision and recall, underscoring its overall robustness. Additionally, the CNN-SVM model achieves an accuracy of 0.946045, reflecting its strong overall performance in classifying both fraud and non-fraud cases.

**Table 7:** Descriptive statistics of model performance metrics.

Model	Metric	Count	Mean	Std	Median	Max	Min
CNN-SVM	Precision	1000	0.947974	0.006803	0.94863	0.965054	0.920581
	Recall	1000	0.946045	0.006942	0.946455	0.963811	0.917651
	F1-Score	1000	0.945986	0.006956	0.94641	0.963787	0.917508
	Accuracy	1000	0.946045	0.006942	0.946455	0.963811	0.917651
LR	Precision	1000	0.576526	0.022569	0.576408	0.635524	0.514889
	Recall	1000	0.57621	0.022827	0.576071	0.635524	0.514402
	F1-Score	1000	0.575434	0.023929	0.575787	0.635524	0.510394
	Accuracy	1000	0.57621	0.022827	0.576071	0.635524	0.514402
RF	Precision	1000	0.852326	0.065125	0.856786	0.959606	0.598909
	Recall	1000	0.528773	0.009203	0.529195	0.55608	0.503419
	F1-Score	1000	0.532389	0.01654	0.533498	0.579594	0.484207
	Accuracy	1000	0.914974	0.001883	0.915209	0.919919	0.908479
SVM	Precision	1000	0.537816	0.009674	0.538369	0.570264	0.502217
	Recall	1000	0.533546	0.009878	0.533973	0.5613	0.502216
	F1-Score	1000	0.518872	0.019051	0.525159	0.555248	0.472323
	Accuracy	1000	0.533546	0.009878	0.533973	0.5613	0.502216
XGBoost	Precision	1000	0.771422	0.045906	0.771113	0.924552	0.61495
	Recall	1000	0.56564	0.01457	0.566014	0.623618	0.517927
	F1-Score	1000	0.591222	0.021834	0.591749	0.668832	0.515098
	Accuracy	1000	0.915898	0.003433	0.915882	0.926649	0.905114

In contrast, the Logistic Regression (LR) model shows subpar results. Both its precision and recall hover around 0.576, demonstrating its limited ability to accurately identify fraudulent cases. With similarly low accuracy, LR proves to be unsuitable for this task, failing to capture enough fraudulent instances [42].

The Random Forest (RF) model performs relatively well in terms of accuracy, achieving 0.914974, but its recall is notably low at 0.528773. This suggests that while RF can classify non-fraudulent cases well, it misses many fraud cases, making it less effective for detecting fraud. The precision of 0.852326 is relatively high, but the low recall indicates an imbalance in its predictions, where false negatives are more common.

The Support Vector Machine (SVM) model struggles significantly, with both precision (0.537816) and recall (0.533546) falling well below the performance of other models. These low scores suggest that SVM is less capable of distinguishing fraudulent cases, likely due to its inability to handle the complexities and imbalances inherent in the dataset.

Lastly, the XGBoost model shows promising precision (0.771422) and accuracy (0.915898), but its recall (0.56564) and F1-score (0.591222) are lower than those of the CNN-SVM model. This indicates that while XGBoost performs well overall, it has some limitations in capturing all instances of fraud, highlighting areas for potential improvement in fraud detection.

Overall, the CNN-SVM model stands out as the most effective at identifying fraudulent cases with high precision, recall, and accuracy, making it the best-performing model in this comparison.

#### 4.3.2. Analysis of Variance (ANOVA).

As shown in Table 8, the normality test assesses whether the distribution of performance metrics (precision, recall, F1-score, and accuracy) follows a normal distribution. The results reveal that most models exhibit p-values less than 0.05, indicating that their performance metrics do not adhere to a normal distribution. Specifically, the CNN-SVM, LR, RF, and SVM models show extremely low p-values (near zero), suggesting significant deviations from normality. However, the XGBoost model shows higher p-values for precision and recall (0.173655 and 0.841317, respectively), implying that these metrics might be approximately normally distributed for this model. Given that the normality assumption is violated for most models, parametric tests like ANOVA are not entirely appropriate for drawing conclusions.

**Table 8:** Normality test results for the performance metrics of the compared models.

Metric	Test	Statistic	p value
Precision	ANOVA	22224.84	0
	CNN-SVM Normality	–	0
	LR Normality	–	0
	XGBoost Normality	–	0.173655
	RF Normality	–	0
	SVM Normality	–	0.103567
	Levene’s Test	748.2667	0
Recall	ANOVA	164020.5	0
	CNN-SVM Normality	–	0.000001
	LR Normality	–	0
	XGBoost Normality	–	0.841317
	RF Normality	–	0.000004
	SVM Normality	–	0.018968
	Levene’s Test	243.532	0
F1-Score	ANOVA	90937.74	0
	CNN-SVM Normality	–	0.000001
	LR Normality	–	0
	XGBoost Normality	–	0.330066
	RF Normality	–	0.000006
	SVM Normality	–	0
	Levene’s Test	167.997	0
Accuracy	ANOVA	305072.9	0
	CNN-SVM Normality	–	0.000001
	LR Normality	–	0
	XGBoost Normality	–	0.002848
	RF Normality	–	0
	SVM Normality	–	0.018968
	Levene’s Test	613.0855	0

Levene’s test, which assesses the homogeneity of variances across the models, tests the null hypothesis that the variances are equal. The results for all performance metrics (precision, recall, F1-score, and accuracy) show very small p-values (0.0000), indicating significant differences in variances among the models. This result further emphasizes the need to apply non-parametric tests, as the unequal variances violate the assumptions of ANOVA.

The ANOVA results, which examine the null hypothesis that there are no significant differences in the models’ performances, reveal extremely small p-values (0.0000) for all metrics, leading to the rejection of the null hypothesis. This suggests that, overall, the performance of at least one model is significantly different from the others. However, due to the violations of normality and homogeneity of variance assumptions, the ANOVA results should be interpreted with caution. Therefore, alternative statistical approaches, like the Kruskal-Wallis H test, are considered more reliable.

#### 4.3.3. Kruskal‒Wallis H test and bootstrap test.

In the context of financial fraud detection, the Kruskal-Wallis H test results presented in Table 9 demonstrate that there are statistically significant differences in the distribution of performance metrics (Precision, Recall, F1-Score, and Accuracy) across the various models. The p-values for all metrics are reported as 0, which indicates strong evidence that the models’ performances differ significantly. This suggests that the selection of the model plays a crucial role in determining the effectiveness of fraud detection. The larger the Kruskal-Wallis H statistic, the greater the disparity in model performance, and thus the more critical it is to choose an appropriate model for the task at hand.

**Table 9:** Results of the Kruskal–Wallis H test for model performance metrics.

Metric	Kruskal‒Wallis H Statistic	Kruskal‒Wallis H p value
Precision	4487.223822	0
Recall	4036.166978	0
F1-Score	4115.694605	0
Accuracy	4503.647294	0

To further support this conclusion, the bootstrap results presented in Table 10 provide confidence intervals (CIs) for the performance metrics of each model. The CNN-SVM consistently shows the highest CIs across all metrics, indicating that its performance is not only the best among the models but also the most consistent. The relatively narrow confidence intervals for CNN-SVM across all four metrics (Precision, Recall, F1-Score, and Accuracy) further confirm its robustness and reliability in predicting fraudulent activities.

**Table 10:** Bootstrap confidence intervals for the performance metrics of the compared models.

Metric	Metric Type	CI Lower	CI Upper
Precision	CNN-SVM	0.94758645	0.948379702
	LR	0.57519537	0.577972974
	XGBoost	0.7683982	0.774233877
	RF	0.8483712	0.856171826
	SVM	0.53722831	0.538440177
Recall	CNN-SVM	0.945613	0.946482395
	LR	0.57489113	0.577571704
	XGBoost	0.56465198	0.566603961
	RF	0.52820411	0.529318046
	SVM	0.5329457	0.534169295
F1-Score	CNN-SVM	0.94556941	0.946400382
	LR	0.5738399	0.576961469
	XGBoost	0.58982924	0.592574701
	RF	0.53141864	0.533332282
	SVM	0.51769802	0.520015727
Accuracy	CNN-SVM	0.94559744	0.946471196
	LR	0.57486068	0.577520052
	XGBoost	0.91568097	0.916104997
	RF	0.91486003	0.915090848
	SVM	0.53297375	0.534186568

For instance, the precision of CNN-SVM ranges from 0.9476 to 0.9484, while its recall ranges from 0.9456 to 0.9465. These narrow intervals suggest that CNN-SVM’s predictions are highly reliable and stable. In contrast, the precision of Logistic Regression (LR) ranges from 0.5752 to 0.5780, and its recall ranges from 0.5749 to 0.5776, indicating less consistency and lower performance in detecting fraud.

The bootstrap test, which involves repeated sampling to estimate the confidence intervals for a model’s performance statistics, highlights the superior consistency of CNN-SVM compared to other models. While models like XGBoost and Random Forest (RF) demonstrate good performance in some areas, their confidence intervals are generally wider, suggesting a higher degree of uncertainty in their predictions. For example, XGBoost’s precision ranges from 0.7684 to 0.7742, and RF’s precision ranges from 0.8484 to 0.8562. These wider intervals indicate more variability in their performance compared to CNN-SVM.

In contrast, Logistic Regression (LR) and Support Vector Machine (SVM) perform significantly poorer across all metrics. The confidence intervals for LR and SVM are notably lower, especially in Precision, Recall, and F1-Score, indicating that these models are not as effective at detecting fraud cases. For example, SVM’s precision ranges from 0.5372 to 0.5384, and its recall ranges from 0.5329 to 0.5342, showing lower and less consistent performance.

## 5. Discussion

The CNN-SVM model significantly improves financial fraud detection, particularly for Chinese listed companies, by offering high accuracy and reliability. Through extensive statistical analysis, including descriptive statistics, ANOVA, Kruskal-Wallis H test, and bootstrap testing, the model shows superior performance compared to other models like Logistic Regression, Random Forest, SVM, and XGBoost. It excels in key metrics such as precision, recall, F1-score, and accuracy, consistently identifying fraudulent cases with stability across multiple tests. The model’s narrow confidence intervals, indicated by bootstrap results, highlight its reliability and ability to minimize false positives and false negatives. CNN-SVM outperforms other models, especially in handling complex fraud detection tasks, offering clear advantages in both precision and recall. Statistical tests confirm its superiority in all metrics. Its ability to capture most fraud cases while minimizing false positives makes it the most effective tool for financial fraud detection in this study.

The CNN-SVM model for detecting financial fraud can significantly enhance the effectiveness of financial management and regulatory practices. By more accurately identifying financial fraud behaviors, the model can help regulatory agencies and companies detect potential risks early and improve fraud detection processes, thereby reducing economic losses and maintaining market stability. In addition, the model’s high accuracy and good generalization ability can enhance the timeliness and efficiency of regulation, making financial management more scientific and effective [34].

In terms of practical applications, integrating this CNN-SVM model into regulatory frameworks could significantly enhance the efficiency and effectiveness of financial fraud detection. The model could be employed by financial institutions or regulatory bodies to proactively monitor and flag suspicious activities, enabling quicker responses to potential fraudulent behavior. Furthermore, it could be embedded into automated systems for continuous surveillance, offering real-time alerts and facilitating timely interventions to prevent or minimize financial damage. The empirical results suggest that combining deep representation learning with margin-based classification is a promising direction for listed-company financial fraud detection. Although the computational cost of CNN-SVM is higher than that of traditional models, its inference speed remains within a practically acceptable range for financial risk screening. In batch-processing scenarios, such as annual-report screening, the CNN-SVM model is capable of processing a substantial number of firm-level observations within a limited time window. This performance fully meets the requirements of offline analysis, efficiently processing large amounts of data while ensuring timely and accurate analysis. In near-real-time monitoring scenarios, such as quarterly report processing, assuming 10,000 company data entries need to be processed daily, the total processing time for CNN-SVM is about 12.4 seconds, which is much lower than the time required for manual review. This greatly improves processing efficiency, reducing both labor costs and time consumption. Therefore, the proposed model appears suitable for offline screening and periodic monitoring tasks, but it should be regarded as a screening tool rather than a standalone adjudication mechanism. In practical use, the proposed model should be positioned as a screening and prioritization tool rather than a fully autonomous decision-making system.

Despite its practical value, the model still faces several methodological and deployment-related challenges. First, false-positive predictions may create reputational, legal, and supervisory risks if a firm is incorrectly flagged as potentially fraudulent. The reputational damage that could result from a company being wrongly classified as engaging in fraudulent behavior, which may lead to a loss of investor confidence and public trust. Such errors could severely impact the company’s financial standing and lead to legal consequences, even when no fraudulent activity has taken place. Moreover, the use of machine-learning models in fraud detection raises important concerns regarding fairness, transparency, and explainability. For instance, if the model’s features are biased or not representative of the true financial activities of a company, it could disproportionately target certain firms or sectors, undermining the integrity of the detection process.

Second, practical deployment also depends on data quality, periodic model updating, and the stability of the model across industries and reporting periods. One of the main challenges lies in ensuring the quality and comprehensiveness of the training data. The model relies heavily on historical data, which may not always capture the evolving nature of fraudulent tactics. Because the current dataset is drawn from the Chinese market, the model’s transferability to other markets remains to be validated in future studies. Additionally, the inherently imbalanced nature of fraud detection datasets makes minority-class learning difficult and may affect the robustness of model performance across different samples. Although oversampling is useful for improving minority-class detection, it may also amplify duplicated patterns and thus affect out-of-sample generalizability.

## 6. Conclusions

In this paper, a hybrid CNN-SVM model was developed and evaluated for financial fraud detection among Chinese non-financial A-share listed companies. The results show that, compared with the benchmark models, the CNN-based hybrid models performed better in reducing false negatives and achieved superior overall classification performance. In particular, the CNN-SVM model achieved an AUC of 0.97, a recall of 0.99, a precision of 0.96, and an F1-score of 0.97, indicating strong detection performance within the present experimental setting. Furthermore, statistical comparison with RF, XGBoost, LR, and SVM suggests that the proposed CNN-SVM model performs significantly better than the benchmark models across the reported evaluation metrics.

Despite the encouraging results of this study, several limitations should be acknowledged. First, from a data perspective, the selection of variables inevitably involves a degree of subjectivity. Although this study selected 87 fraud-identification indicators from corporate governance, financial supervision, financial indicators, and business operations, these variables cannot cover all information potentially relevant to fraud. Moreover, the current feature system is primarily quantitative and does not yet incorporate textual disclosures or other unstructured information, while reliance on publicly available data means that some relevant fraud-related information remains unobservable. Second, from a model perspective, the performance of the CNN-SVM framework still depends heavily on the quality, representativeness, and size of the training data. In addition, class imbalance may affect model stability, and the use of oversampling can improve minority-class learning while also introducing a potential risk to out-of-sample generalizability. Although early stopping was used to mitigate overfitting, the model may still perform less robustly on unseen data than on the development data.

Future research can focus on the following areas: First, future studies can establish industry-specific fraud-identification systems by incorporating sectoral characteristics into indicator design, such as emphasizing goodwill and R&D capitalization risks in technology- and pharmaceutical-related industries and placing greater attention on construction-in-progress and leverage-related risks in the construction industry. Second, future studies may integrate textual risk disclosures, regulatory punishment reports, and other unstructured information with structured financial indicators to improve fraud detection performance and early-warning capability [43]. Third, future studies can explore other deep-learning and hybrid architectures while also improving model interpretability through explainable AI techniques such as SHAP and LIME [44,45], so as to quantify the contribution of individual variables to firm-level fraud-risk predictions. Fourth, future research can establish a continuous monitoring and updating mechanism for the model, expand validation to other capital markets, and examine the model’s performance under streaming or periodically updated data environments. Such extensions would further improve the practical value, interpretability, and external validity of financial fraud detection models.

## Supporting information

S1 Fig Feature Variable Classification. (DOCX)

S1 Table Fraudulent Sample Feature Value Statistics. (DOCX)

S2 Table Non-Fraudulent Sample Feature Value Statistics. (DOCX)

S1 Data Dataset used in this study. (DOCX)

S1 File Detailed Explanation of the CNN-SVM Model Code. (DOCX)
