# Supplementary material for: Research on a financial fraud identification model by fusing a convolutional neural network
Source: PLoS One. 2026 May 22;21(5):e0348569. doi: 10.1371/journal.pone.0348569 (PMC13196949; doi:10.1371/journal.pone.0348569)

**S1 Fig. Feature Variable Classification.** This paper systematically selects feature variables across four dimensions: corporate governance, financial supervision, financial indicators, and corporate operations, to comprehensively assess the financial fraud risk of enterprises. First, in terms of corporate governance, the analysis focuses on board structure, shareholder rights, and management characteristics to reveal the effectiveness of management efficiency and internal checks and balances.

Second, the role of financial supervision is crucial; by examining indicators such as audit quality and information disclosure transparency, it ensures the accuracy of financial reports and reduces the likelihood of fraud. Third, financial indicators directly reflect the financial health of enterprises. This paper constructs a complete financial analysis system through multiple dimensions, including profitability and solvency, to help identify potential fraudulent activities. Finally, abnormal behaviors at the operational level may also conceal financial fraud risks. This paper addresses areas such as market valuation and investment management, establishing a detailed analytical framework with specific indicators to effectively identify and prevent financial fraud risks.


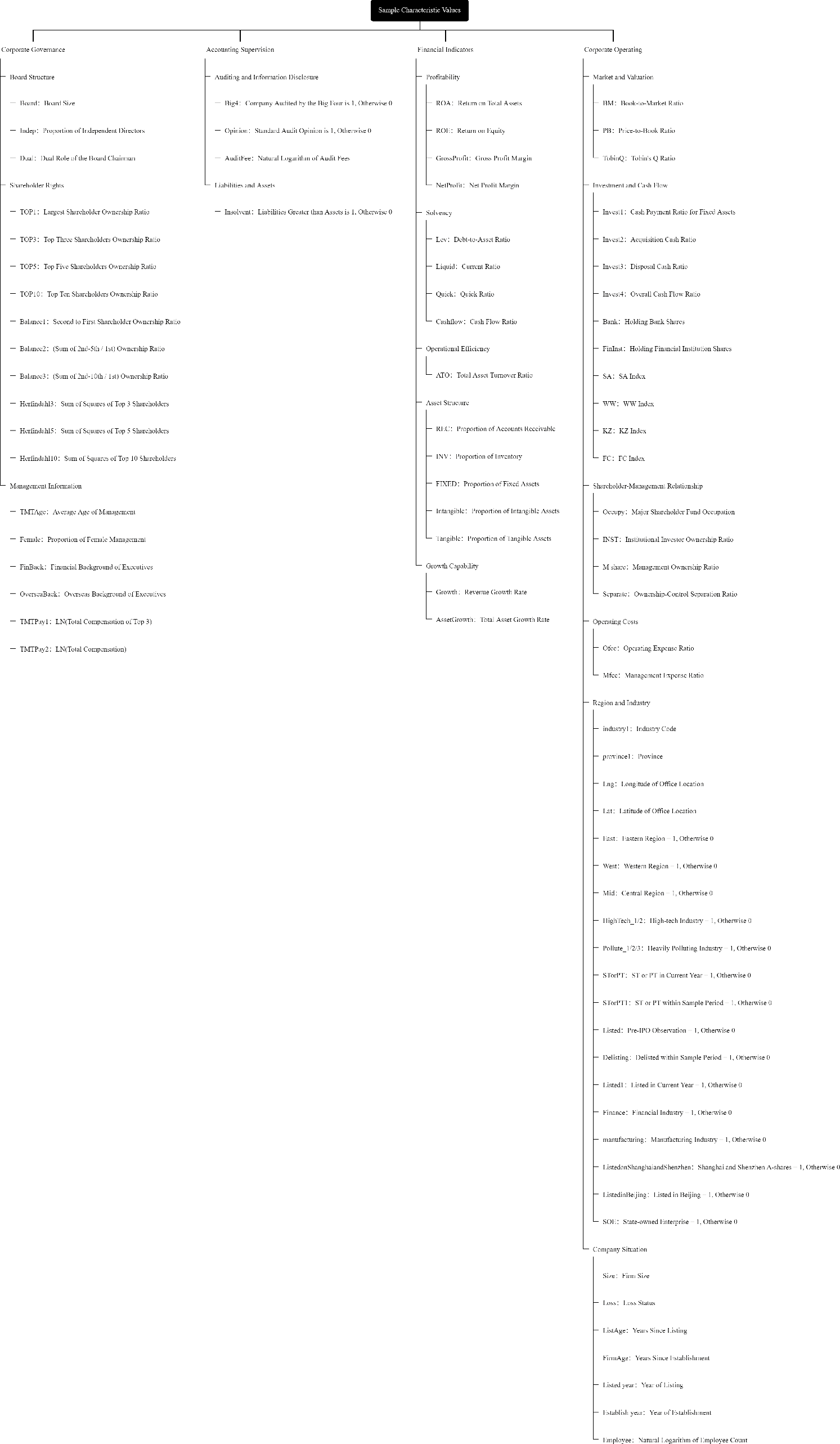

Supplement: S1 Fig — (DOCX) [file pone.0348569.s001.docx]
