# Supplementary material for: Research on a financial fraud identification model by fusing a convolutional neural network
Source: PLoS One. 2026 May 22;21(5):e0348569. doi: 10.1371/journal.pone.0348569 (PMC13196949; doi:10.1371/journal.pone.0348569)
Supplement: S1 Table — (DOCX) [file pone.0348569.s002.docx]

**S1 Table. Fraudulent Sample Feature Value Statistics.** This table presents the statistical analysis of feature values for fraudulent samples, including the symbols of various variables, sample size, mean, standard deviation, minimum, median, and maximum values. Through these statistical data, we gain deeper insights into the distribution of fraudulent samples across different feature variables. These feature values not only reflect the potential characteristics of fraudulent behavior but also provide foundational data for subsequent analyses and comparisons, aiding in the identification of key factors associated with fraud.

|  |  | Variable Symbols | Sample Size | Mean | Standard Deviation | Min. | Med | Max |
| --- | --- | --- | --- | --- | --- | --- | --- | --- |
| Corporate Governance | Board Structure | Board | 659 | 2.13 | 0.22 | 1.61 | 2.2 | 2.71 |
|  |  | Indep | 659 | 37.8 | 5.84 | 25 | 36.36 | 60 |
|  |  | Dual | 659 | 0.25 | 0.43 | 0 | 0 | 1 |
|  | Shareholder Rights | TOP1 | 659 | 32.73 | 15.15 | 8.38 | 30.66 | 75.84 |
|  |  | TOP3 | 659 | 44.93 | 14.77 | 15.68 | 44.11 | 87.49 |
|  |  | TOP5 | 659 | 49.18 | 14.91 | 17.72 | 49.48 | 88.87 |
|  |  | TOP10 | 659 | 53.91 | 15.38 | 20.84 | 54.35 | 90.68 |
|  |  | Balance1 | 659 | 0.35 | 0.31 | 0.01 | 0.22 | 1 |
|  |  | Balance2 | 659 | 0.71 | 0.68 | 0.02 | 0.47 | 2.91 |
|  |  | Balance3 | 659 | 0.93 | 0.88 | 0.03 | 0.64 | 4.17 |
|  |  | Herfindahl3 | 659 | 0.14 | 0.11 | 0.01 | 0.11 | 0.58 |
|  |  | Herfindahl5 | 659 | 0.15 | 0.11 | 0.01 | 0.11 | 0.58 |
|  |  | Herfindahl10 | 659 | 0.15 | 0.11 | 0.01 | 0.11 | 0.58 |
|  | Management Information | TMTAge | 659 | 48.38 | 3.03 | 40.75 | 48.69 | 56.43 |
|  |  | Female | 659 | 18.06 | 10.87 | 0 | 17.65 | 50 |
|  |  | FinBack | 659 | 0.7 | 0.46 | 0 | 1 | 1 |
|  |  | OverseaBack | 659 | 0.51 | 0.5 | 0 | 1 | 1 |
|  |  | TMTPay1 | 659 | 14.2 | 0.69 | 12.38 | 14.19 | 16.75 |
|  |  | TMTPay2 | 659 | 14.98 | 0.74 | 12.82 | 14.99 | 17.49 |
| Accounting Supervision | Auditing and Information Disclosure | Big4 | 659 | 0.02 | 0.15 | 0 | 0 | 1 |
|  |  | Opinion | 659 | 0.91 | 0.29 | 0 | 1 | 1 |
|  |  | AuditFee | 659 | 13.56 | 0.6 | 12.1 | 13.46 | 15.93 |
|  | Liabilities and Assets | Insolvent | 659 | 0 | 0 | 0 | 0 | 0 |
| Financial Indicators | Profitability | ROA | 659 | 0.02 | 0.07 | -0.33 | 0.03 | 0.22 |
|  |  | ROE | 659 | 0.03 | 0.15 | -0.81 | 0.05 | 0.42 |
|  |  | GrossProfit | 659 | 0.27 | 0.18 | -0.04 | 0.23 | 0.82 |
|  |  | NetProfit | 659 | 0.04 | 0.19 | -1.32 | 0.05 | 0.54 |
|  | Solvency | Lev | 659 | 0.49 | 0.21 | 0.05 | 0.51 | 0.89 |
|  |  | Liquid | 659 | 2.07 | 2.41 | 0.24 | 1.38 | 19.37 |
|  |  | Quick | 659 | 1.61 | 2.16 | 0.13 | 0.95 | 17.08 |
|  |  | Cashflow | 659 | 0.03 | 0.07 | -0.2 | 0.03 | 0.26 |
|  | Operational Efficiency | ATO | 659 | 0.62 | 0.43 | 0.06 | 0.52 | 3.02 |
|  | Asset Structure | REC | 659 | 0.11 | 0.1 | 0 | 0.09 | 0.51 |
|  |  | INV | 659 | 0.15 | 0.15 | 0 | 0.11 | 0.77 |
|  |  | FIXED | 659 | 0.22 | 0.18 | 0 | 0.18 | 0.77 |
|  |  | Intangible | 659 | 0.05 | 0.05 | 0 | 0.03 | 0.33 |
|  |  | Tangible | 659 | 0.91 | 0.1 | 0.47 | 0.95 | 1 |
|  | Growth Capability | Growth | 659 | 0.18 | 0.52 | -0.61 | 0.08 | 3.81 |
|  |  | AssetGrowth | 659 | 0.18 | 0.42 | -0.38 | 0.09 | 3.92 |
| Corporate Operating | Market and Valuation | BM | 659 | 0.59 | 0.25 | 0.09 | 0.59 | 1.2 |
|  |  | PB | 659 | 4.27 | 4.04 | 0.59 | 2.98 | 44.5 |
|  |  | TobinQ | 659 | 2.18 | 1.48 | 0.83 | 1.68 | 11.7 |
|  | Investment and Cash Flow | Invest1 | 659 | 0.05 | 0.06 | 0 | 0.04 | 0.39 |
|  |  | Invest2 | 659 | 0.07 | 0.08 | 0 | 0.05 | 0.64 |
|  |  | Invest3 | 659 | 0.05 | 0.06 | -0.03 | 0.03 | 0.39 |
|  |  | Invest4 | 659 | 0.06 | 0.08 | -0.1 | 0.04 | 0.6 |
|  |  | Bank | 659 | 0.16 | 0.37 | 0 | 0 | 1 |
|  |  | FinInst | 659 | 0.13 | 0.33 | 0 | 0 | 1 |
|  |  | SA | 659 | -3.77 | 0.25 | -4.53 | -3.76 | -3.1 |
|  |  | WW | 659 | -1.01 | 0.07 | -1.26 | -1.01 | -0.59 |
|  |  | KZ | 659 | 2.04 | 2.09 | -6.34 | 2.23 | 7.18 |
|  |  | FC | 659 | 0.42 | 0.28 | 0 | 0.39 | 0.97 |
|  | Shareholder-Management Relationship | Occupy | 659 | 0.02 | 0.03 | 0 | 0.01 | 0.21 |
|  |  | INST | 659 | 43.84 | 23.89 | 0.16 | 44.41 | 120.52 |
|  |  | M share | 659 | 10.26 | 17.81 | 0 | 0.12 | 70.17 |
|  |  | Separate | 659 | 5.16 | 7.5 | -1.87 | 0.14 | 28.82 |
|  | Operating Costs | Ofee | 659 | 0.18 | 0.15 | 0.01 | 0.13 | 0.79 |
|  |  | Mfee | 659 | 0.1 | 0.09 | 0.01 | 0.08 | 0.64 |
|  | Region and Industry | industry1 | 659 | 40.24 | 18.73 | 1 | 38 | 90 |
|  |  | province1 | 659 | 351320.18 | 124000 | 110000 | 330000 | 650000 |
|  |  | Lng | 659 | 115.92 | 6.91 | 84.88 | 117.21 | 127.18 |
|  |  | Lat | 659 | 31.92 | 6.18 | 19.99 | 31.24 | 45.77 |
|  |  | East | 659 | 0.68 | 0.47 | 0 | 1 | 1 |
|  |  | West | 659 | 0.16 | 0.36 | 0 | 0 | 1 |
|  |  | Mid | 659 | 0.17 | 0.38 | 0 | 0 | 1 |
|  |  | HighTech_1 | 659 | 0.58 | 0.49 | 0 | 1 | 1 |
|  |  | HighTech_2 | 659 | 0.46 | 0.5 | 0 | 0 | 1 |
|  |  | Pollute_1 | 659 | 0.26 | 0.44 | 0 | 0 | 1 |
|  |  | Pollute_2 | 659 | 0.34 | 0.48 | 0 | 0 | 1 |
|  |  | Pollute_3 | 659 | 0.36 | 0.48 | 0 | 0 | 1 |
|  |  | STorPT | 659 | 0 | 0 | 0 | 0 | 0 |
|  |  | STorPT1 | 659 | 0.31 | 0.46 | 0 | 0 | 1 |
|  |  | Listed | 659 | 0 | 0 | 0 | 0 | 0 |
|  |  | Delisting | 659 | 0 | 0 | 0 | 0 | 0 |
|  |  | Listed1 | 659 | 0.01 | 0.1 | 0 | 0 | 1 |
|  |  | Finance | 659 | 0 | 0 | 0 | 0 | 0 |
|  |  | manufacturing | 659 | 0.58 | 0.49 | 0 | 1 | 1 |
|  |  | ListedonShanghaiandShenzhen | 659 | 1 | 0 | 1 | 1 | 1 |
|  |  | ListedinBeijing | 659 | 0 | 0 | 0 | 0 | 0 |
|  |  | SOE | 659 | 0.36 | 0.48 | 0 | 0 | 1 |
|  | Company Situation | Size | 659 | 22.07 | 1.19 | 19.51 | 22.01 | 26.43 |
|  |  | Loss | 659 | 0.15 | 0.36 | 0 | 0 | 1 |
|  |  | ListAge | 659 | 2.2 | 0.74 | 0 | 2.4 | 3.33 |
|  |  | FirmAge | 659 | 2.83 | 0.35 | 1.1 | 2.83 | 3.61 |
|  |  | Listed year | 659 | 2003.46 | 6.91 | 1990 | 2002 | 2018 |
|  |  | Establish year | 659 | 1996.76 | 5.43 | 1981 | 1998 | 2009 |
|  |  | Employee | 659 | 7.54 | 1.29 | 3.56 | 7.56 | 11.14 |
