# Supplementary material for: Research on a financial fraud identification model by fusing a convolutional neural network
Source: PLoS One. 2026 May 22;21(5):e0348569. doi: 10.1371/journal.pone.0348569 (PMC13196949; doi:10.1371/journal.pone.0348569)
Supplement: S2 Table — (DOCX) [file pone.0348569.s003.docx]

**S2 Table. Non-Fraudulent Sample Feature Value Statistics.** This table presents the statistical analysis of feature values for non-fraudulent samples, also including the symbols of variables, sample size, mean, standard deviation, minimum, median, and maximum values. By analyzing the feature values of non-fraudulent samples, we can identify the characteristics of enterprises operating under normal conditions. This data provides a basis for comparing fraudulent and non-fraudulent samples, helping to reveal the differences between fraudulent behavior and normal operations, thereby offering important references for subsequent research.

|  |  | Variable Symbols | Sample Size | Mean | Standard Deviation | Minimum | Median | Maximum |
| --- | --- | --- | --- | --- | --- | --- | --- | --- |
| Corporate Governance | Board Structure | Board | 6770 | 2.17 | 0.2 | 1.61 | 2.2 | 2.71 |
|  |  | Indep | 6770 | 37.03 | 5.27 | 25 | 33.33 | 60 |
|  |  | Dual | 6770 | 0.19 | 0.39 | 0 | 0 | 1 |
|  | Shareholder Rights | TOP1 | 6770 | 36.67 | 15.01 | 8.09 | 35.02 | 75.84 |
|  |  | TOP3 | 6770 | 48.86 | 15.23 | 15.13 | 48.68 | 87.84 |
|  |  | TOP5 | 6770 | 52.63 | 15.22 | 17.72 | 52.55 | 89.11 |
|  |  | TOP10 | 6770 | 56.94 | 15.23 | 20.84 | 57.4 | 90.97 |
|  |  | Balance1 | 6770 | 0.3 | 0.28 | 0.01 | 0.19 | 1 |
|  |  | Balance2 | 6770 | 0.59 | 0.56 | 0.02 | 0.4 | 2.84 |
|  |  | Balance3 | 6770 | 0.75 | 0.72 | 0.03 | 0.53 | 4.45 |
|  |  | Herfindahl3 | 6770 | 0.17 | 0.12 | 0.01 | 0.14 | 0.58 |
|  |  | Herfindahl5 | 6770 | 0.17 | 0.12 | 0.01 | 0.15 | 0.58 |
|  |  | Herfindahl10 | 6770 | 0.17 | 0.12 | 0.01 | 0.15 | 0.58 |
|  | Management Information | TMTAge | 6770 | 48.88 | 3.17 | 39.69 | 48.91 | 57.79 |
|  |  | Female | 6770 | 16.35 | 10.23 | 0 | 15 | 53.85 |
|  |  | FinBack | 6770 | 0.68 | 0.47 | 0 | 1 | 1 |
|  |  | OverseaBack | 6770 | 0.49 | 0.5 | 0 | 0 | 1 |
|  |  | TMTPay1 | 6770 | 14.24 | 0.74 | 11.78 | 14.22 | 16.88 |
|  |  | TMTPay2 | 6770 | 15.03 | 0.79 | 12.39 | 15 | 17.57 |
| Accounting Supervision | Auditing and Information Disclosure | Big4 | 6770 | 0.06 | 0.25 | 0 | 0 | 1 |
|  |  | Opinion | 6770 | 0.98 | 0.13 | 0 | 1 | 1 |
|  |  | AuditFee | 6770 | 13.54 | 0.71 | 12.1 | 13.38 | 16.06 |
|  | Liabilities and Assets | Insolvent | 6770 | 0 | 0 | 0 | 0 | 0 |
| Financial Indicators | Profitability | ROA | 6770 | 0.04 | 0.05 | -0.33 | 0.04 | 0.26 |
|  |  | ROE | 6770 | 0.08 | 0.11 | -0.74 | 0.08 | 0.47 |
|  |  | GrossProfit | 6770 | 0.28 | 0.17 | -0.06 | 0.24 | 0.85 |
|  |  | NetProfit | 6770 | 0.08 | 0.13 | -1.26 | 0.07 | 0.6 |
|  | Solvency | Lev | 6770 | 0.46 | 0.21 | 0.03 | 0.47 | 0.91 |
|  |  | Liquid | 6770 | 2.39 | 3.08 | 0.24 | 1.47 | 35.5 |
|  |  | Quick | 6770 | 1.87 | 2.85 | 0.13 | 0.99 | 33.96 |
|  |  | Cashflow | 6770 | 0.04 | 0.07 | -0.22 | 0.04 | 0.28 |
|  | Operational Efficiency | ATO | 6770 | 0.68 | 0.49 | 0.06 | 0.56 | 3.11 |
|  | Asset Structure | REC | 6770 | 0.1 | 0.1 | 0 | 0.07 | 0.49 |
|  |  | INV | 6770 | 0.17 | 0.16 | 0 | 0.12 | 0.77 |
|  |  | FIXED | 6770 | 0.23 | 0.18 | 0 | 0.19 | 0.77 |
|  |  | Intangible | 6770 | 0.05 | 0.05 | 0 | 0.03 | 0.38 |
|  |  | Tangible | 6770 | 0.94 | 0.08 | 0.45 | 0.96 | 1 |
|  | Growth Capability | Growth | 6770 | 0.19 | 0.45 | -0.66 | 0.12 | 4.02 |
|  |  | AssetGrowth | 6770 | 0.21 | 0.43 | -0.37 | 0.11 | 5.12 |
| Corporate Operating | Market and Valuation | BM | 6770 | 0.61 | 0.24 | 0.06 | 0.6 | 1.25 |
|  |  | PB | 6770 | 3.79 | 3.46 | 0.41 | 2.82 | 44.5 |
|  |  | TobinQ | 6770 | 2.08 | 1.42 | 0.8 | 1.67 | 15.61 |
|  | Investment and Cash Flow | Invest1 | 6770 | 0.07 | 0.07 | 0 | 0.04 | 0.52 |
|  |  | Invest2 | 6770 | 0.08 | 0.09 | 0 | 0.05 | 2.38 |
|  |  | Invest3 | 6770 | 0.06 | 0.07 | -0.05 | 0.04 | 0.52 |
|  |  | Invest4 | 6770 | 0.07 | 0.1 | -0.11 | 0.05 | 2.94 |
|  |  | Bank | 6770 | 0.19 | 0.39 | 0 | 0 | 1 |
|  |  | FinInst | 6770 | 0.17 | 0.37 | 0 | 0 | 1 |
|  |  | SA | 6770 | -3.7 | 0.22 | -4.56 | -3.7 | -2.95 |
|  |  | WW | 6770 | -1.01 | 0.08 | -1.26 | -1.01 | -0.59 |
|  |  | KZ | 6770 | 1.7 | 2.17 | -8.71 | 1.9 | 7.18 |
|  |  | FC | 6770 | 0.44 | 0.29 | 0 | 0.42 | 0.98 |
|  | Shareholder-Management Relationship | Occupy | 6770 | 0.02 | 0.02 | 0 | 0.01 | 0.21 |
|  |  | INST | 6770 | 51.69 | 24.11 | 0.12 | 53.45 | 121.81 |
|  |  | M share | 6770 | 8.87 | 17.54 | 0 | 0.01 | 70.6 |
|  |  | Separate | 6770 | 4.85 | 7.67 | -10.32 | 0 | 30.25 |
|  | Operating Costs | Ofee | 6770 | 0.16 | 0.13 | 0.01 | 0.13 | 0.79 |
|  |  | Mfee | 6770 | 0.1 | 0.08 | 0.01 | 0.08 | 0.64 |
|  | Region and Industry | industry1 | 6770 | 41.29 | 18.97 | 1 | 38 | 90 |
|  |  | province1 | 6770 | 335129.99 | 134000 | 110000 | 330000 | 650000 |
|  |  | Lng | 6770 | 116 | 6.88 | 80.2 | 117.12 | 131.02 |
|  |  | Lat | 6770 | 32.72 | 5.76 | 19.28 | 31.25 | 47.21 |
|  |  | East | 6770 | 0.67 | 0.47 | 0 | 1 | 1 |
|  |  | West | 6770 | 0.18 | 0.38 | 0 | 0 | 1 |
|  |  | Mid | 6770 | 0.15 | 0.35 | 0 | 0 | 1 |
|  |  | HighTech_1 | 6770 | 0.52 | 0.5 | 0 | 1 | 1 |
|  |  | HighTech_2 | 6770 | 0.39 | 0.49 | 0 | 0 | 1 |
|  |  | Pollute_1 | 6770 | 0.23 | 0.42 | 0 | 0 | 1 |
|  |  | Pollute_2 | 6770 | 0.29 | 0.46 | 0 | 0 | 1 |
|  |  | Pollute_3 | 6770 | 0.33 | 0.47 | 0 | 0 | 1 |
|  |  | STorPT | 6770 | 0 | 0 | 0 | 0 | 0 |
|  |  | STorPT1 | 6770 | 0.2 | 0.4 | 0 | 0 | 1 |
|  |  | Listed | 6770 | 0 | 0 | 0 | 0 | 0 |
|  |  | Delisting | 6770 | 0 | 0 | 0 | 0 | 0 |
|  |  | Listed1 | 6770 | 0.02 | 0.13 | 0 | 0 | 1 |
|  |  | Finance | 6770 | 0 | 0 | 0 | 0 | 0 |
|  |  | manufacturing | 6770 | 0.58 | 0.49 | 0 | 1 | 1 |
|  |  | ListedonShanghaiandShenzhen | 6770 | 1 | 0.01 | 0 | 1 | 1 |
|  |  | ListedinBeijing | 6770 | 0 | 0.01 | 0 | 0 | 1 |
|  |  | SOE | 6770 | 0.52 | 0.5 | 0 | 1 | 1 |
|  | Company Situation | Size | 6770 | 22.18 | 1.37 | 19.32 | 21.95 | 26.45 |
|  |  | Loss | 6770 | 0.08 | 0.27 | 0 | 0 | 1 |
|  |  | ListAge | 6770 | 2.19 | 0.75 | 0 | 2.4 | 3.4 |
|  |  | FirmAge | 6770 | 2.75 | 0.34 | 0.69 | 2.77 | 3.61 |
|  |  | Listed year | 6770 | 2002.47 | 6.65 | 1990 | 2001 | 2021 |
|  |  | Establish year | 6770 | 1997.06 | 4.78 | 1980 | 1997 | 2014 |
|  |  | Employee | 6770 | 7.7 | 1.38 | 3.56 | 7.67 | 11.18 |
