# Supplementary material for: Research on a financial fraud identification model by fusing a convolutional neural network
Source: PLoS One. 2026 May 22;21(5):e0348569. doi: 10.1371/journal.pone.0348569 (PMC13196949; doi:10.1371/journal.pone.0348569)
Supplement: S1 Data — (DOCX) [file pone.0348569.s004.docx]

**S1 Data. Dataset used in this study.** The data and code in this article are publicly available in a GitHub repository. The public link is as follows: (https://github.com/zhucharlotte/CNNSVM.git). This includes feature data, CNN-SVM model code, feature processing (PCA, correlation, etc.), and code for statistical significance tests.
