# Supplementary material for: Research on a financial fraud identification model by fusing a convolutional neural network
Source: PLoS One. 2026 May 22;21(5):e0348569. doi: 10.1371/journal.pone.0348569 (PMC13196949; doi:10.1371/journal.pone.0348569)
Supplement: S1 File — (DOCX) [file pone.0348569.s005.docx]

**S1 File.Detailed Explanation of the CNN-SVM Model Code**

This code describes a hybrid machine learning model that integrates Convolutional Neural Networks (CNN) with Support Vector Machine (SVM) classifiers to predict a binary target variable, Financialfraud, based on features in the dataset company_data_v2.csv.

**1. Data Loading and Preprocessing**

The first step in the code involves importing necessary libraries and loading the dataset:

data = pd.read_csv('company_data_v2.csv')

Here, the dataset company_data_v2.csv is loaded into a pandas DataFrame. After loading the data, the code cleans the dataset by dropping irrelevant columns (id, year), which do not contribute to the predictive power of the model. The target variable for fraud detection is Financialfraud, where 0 indicates non-fraud and 1 indicates fraud.

data_cleaned = data.drop(['id', 'year'], axis=1)

Next, the class distribution of the target variable is examined:

print("Over-sampling before:")

This step checks the imbalance in the data. Fraud cases (Financialfraud == 1) are typically much fewer than non-fraud cases (Financialfraud == 0), which can lead to model bias towards the majority class. To address this issue, oversampling is performed on the minority class using resample from sklearn's utils:

data_minority_upsampled = resample(data_minority, replace=True, n_samples=len(data_majority), random_state=42)

After oversampling, the dataset becomes balanced, i.e., the number of fraud cases matches the number of non-fraud cases:

print("Over-sampling after:")

**2. Data Splitting and Standardization**

The dataset is split into features (X) and target (y), with X_train and X_test used for training and testing, respectively:

X = data_balanced.drop('Financialfraud', axis=1)

y = data_balanced['Financialfraud']

X_train, X_test, y_train, y_test = train_test_split(X, y, test_size=0.2, random_state=42, stratify=y)

The stratified split ensures that the class distribution in both training and testing sets is similar to that in the full dataset.

To improve the performance of the model, standardization is applied to scale the features. This ensures that all features have a mean of zero and a standard deviation of one, which is critical for many machine learning models, including SVMs:

scaler = StandardScaler()

X_train_scaled = scaler.fit_transform(X_train)

X_test_scaled = scaler.transform(X_test)

**3. Reshaping Data for CNN Input**

Convolutional Neural Networks (CNNs) generally require 3D input data with the shape (samples, time-steps, features). The dataset is reshaped accordingly to match the input requirements of the CNN model:

X_train_reshaped = X_train_scaled.reshape(X_train_scaled.shape[0], X_train_scaled.shape[1], 1)

X_test_reshaped = X_test_scaled.reshape(X_test_scaled.shape[0], X_test_scaled.shape[1], 1)

Here, each sample is reshaped into a 3D tensor where shape[1] is the number of features, and a single channel is used for each sample (1 as the third dimension). This allows CNN to process the features as a sequence of values, even though in this case, they are not time-series data.

**4. Building the CNN Model**

The CNN model is built using Sequential from Keras, with the following layers:

**a. Conv1D Layer:**

Conv1D(filters=32, kernel_size=3, activation='relu', input_shape=(X_train_reshaped.shape[1], 1))

filters=32: Specifies the number of filters (or convolutional kernels) to learn. This layer will learn 32 different feature maps from the input data.

kernel_size=3: The size of the filter, i.e., the length of the 1D convolutional window. This parameter determines the receptive field of the kernel.

activation='relu': The activation function is ReLU, which introduces non-linearity into the model.

input_shape: Defines the input shape to the network (number of features in each sample).

**b. MaxPooling1D Layer:**

MaxPooling1D(pool_size=2)

pool_size=2: This layer performs max pooling, reducing the dimensionality of the feature maps by taking the maximum value over every 2 consecutive values. This helps in down-sampling the data and reducing the computational load.

**c. Flatten Layer:**

Flatten()

This layer flattens the output of the previous layer, transforming the 2D data into a 1D vector that can be fed into the dense layers.

**d. Dense Layers:**

Dense(128, activation='relu')

Dense(1, activation='sigmoid')

The first Dense(128, activation='relu') layer introduces 128 neurons and applies the ReLU activation function to learn non-linear relationships.

The final layer Dense(1, activation='sigmoid') has a single neuron with a sigmoid activation function. This is a binary classification problem, and the sigmoid function outputs a probability between 0 and 1.

The model is compiled with the Adam optimizer, binary cross-entropy loss, and accuracy as the evaluation metric:

model.compile(optimizer='adam', loss='binary_crossentropy', metrics=['accuracy'])

**5. Training the CNN Model**

The CNN model is trained on the reshaped data with a validation split of 0.2 and early stopping to prevent overfitting:

early_stopping = EarlyStopping(monitor='val_loss', patience=3, restore_best_weights=True)

history = model.fit(X_train_reshaped, y_train, epochs=20, batch_size=64, validation_split=0.2, callbacks=[early_stopping])

The early stopping criterion ensures that training stops if the validation loss does not improve for 3 consecutive epochs (patience=3), thus preventing overfitting. The best model weights are restored based on the minimum validation loss.

The training progress, including both training and validation loss, is plotted after training:

plt.plot(history.history['loss'], label='Train Loss')

plt.plot(history.history['val_loss'], label='Validation Loss')

**6. SVM on CNN Output**

After training the CNN model, its output is passed through a Support Vector Machine (SVM) classifier. This is done by using the predictions from the CNN model as features for the SVM:

svm_model = make_pipeline(StandardScaler(), SVC(kernel='linear', probability=True))

svm_model.fit(model.predict(X_train_reshaped), y_train)

The SVC model uses a linear kernel and is trained on the CNN predictions, with the aim of further improving classification accuracy. The output of the CNN model is passed to the SVM classifier, which then makes the final predictions:

y_pred_svm = svm_model.predict(model.predict(X_test_reshaped))

This hybrid CNN-SVM model combines the power of CNN for feature extraction and the SVM's ability to classify based on those extracted features.

**7. Model Evaluation**

Finally, the model's performance can be evaluated using classification metrics such as confusion matrix, classification report, and possibly ROC curves:

classification_report(y_test, y_pred_svm)

confusion_matrix(y_test, y_pred_svm)

These metrics will provide insights into the model's precision, recall, F1-score, and overall accuracy, which are important for assessing performance in imbalanced classification tasks.

**Conclusion**

This hybrid CNN-SVM model is designed to detect financial fraud by combining the strengths of convolutional neural networks (for learning complex patterns in the data) and support vector machines (for effective classification of high-dimensional features). The approach uses data preprocessing steps like standardization and oversampling to mitigate class imbalance and improve model performance. The combination of CNN and SVM offers a powerful tool for tackling fraud detection in financial datasets.
